# State-of-the-Art Deep Learning Methods for Microscopic Image Segmentation: Applications to Cells, Nuclei, and Tissues

**DOI:** 10.3390/jimaging10120311

**Published:** 2024-12-06

**Authors:** Fatma Krikid, Hugo Rositi, Antoine Vacavant

**Affiliations:** 1 Institut Pascal, CNRS, Clermont Auvergne INP, Université Clermont Auvergne, F-63000 Clermont-Ferrand, France; antoine.vacavant@uca.fr; 2 LORIA, CNRS, Université de Lorraine, F-54000 Nancy, France; hugo.rositi@loria.fr

**Keywords:** deep learning, microscopic image, image segmentation, cell segmentation, nucleus segmentation, tissue analysis, biology

## Abstract

Microscopic image segmentation (MIS) is a fundamental task in medical imaging and biological research, essential for precise analysis of cellular structures and tissues. Despite its importance, the segmentation process encounters significant challenges, including variability in imaging conditions, complex biological structures, and artefacts (e.g., noise), which can compromise the accuracy of traditional methods. The emergence of deep learning (DL) has catalyzed substantial advancements in addressing these issues. This systematic literature review (SLR) provides a comprehensive overview of state-of-the-art DL methods developed over the past six years for the segmentation of microscopic images. We critically analyze key contributions, emphasizing how these methods specifically tackle challenges in cell, nucleus, and tissue segmentation. Additionally, we evaluate the datasets and performance metrics employed in these studies. By synthesizing current advancements and identifying gaps in existing approaches, this review not only highlights the transformative potential of DL in enhancing diagnostic accuracy and research efficiency but also suggests directions for future research. The findings of this study have significant implications for improving methodologies in medical and biological applications, ultimately fostering better patient outcomes and advancing scientific understanding.

## 1. Introduction

Microscopic imaging serves as a fundamental tool in both research and diagnosis, particularly within fields like medical science and biology. It offers unparalleled insights into the intricate structures and processes at the cellular and subcellular levels [1]. Segmentation involves categorizing the image pixels based on their respective classes. The primary objective of image segmentation is to group all the pixels within an image [2]. Accurate segmentation of microscopic images is an essential component in the analysis of these images, enabling quantification, classification, and a deeper understanding of the underlying biological components [2]. Additionally, microscopic image segmentation (MIS) not only serves as a fundamental tool in scientific research but also plays a pivotal role in clinical diagnostics. It allows researchers and medical professionals to uncover the intricate world of cells, tissues, and subcellular structures, aiding in the diagnosis of diseases and the advancement of scientific knowledge [3].

Numerous manual segmentation techniques have been proposed, including methods involving feature extraction and region growing [4]. However, relying on manual segmentation, although traditional, presents significant challenges. This method is not only labor-intensive but also susceptible to human bias and inconsistencies. The process is time-consuming, and the precision of outcomes is heavily reliant on the proficiency of the annotators [4]. The infusion of artificial intelligence (AI) holds the potential to mitigate these challenges while augmenting the consistency of segmentation tasks.

Machine learning (ML) has instigated rapid advancements, particularly within the biomedical domain, primarily in the field of image segmentation. Deep learning (DL), a subset of ML with a primary focus on artificial neural networks (ANNs), acts as a driving force behind the increasing expansion of research in imaging sciences and computational pathology [5]. With the rise of DL, particularly the utilization of convolutional neural networks (CNNs) [6], the realm of image segmentation has undergone a profound and transformative evolution. DL algorithms have showcased exceptional capabilities in automating this process, often surpassing traditional methods in terms of precision and efficiency. These progressions have the potential not only to speed up the analysis of microscopic images but also to make the results more accurate [5].

In the field of MIS, significant strides have been made through the evolution of DL architectures. The inception of Fully Convolutional Networks (FCNs) marked a crucial evolution in the field, as it introduced a paradigm shift towards end-to-end pixel-wise segmentation. Its predominant applications were in the segmentation of mitochondria [7] and microvasculature [8]. Building upon this foundation, the subsequent evolution of segmentation methodologies witnessed the emergence and widespread adoption of U-Net architecture [9]. U-Net, characterized by its unique encoder–decoder structure, has demonstrated superior performance in preserving fine-grained details crucial for microscopic imagery. Beginning with U-Net, numerous variants and networks were created for MIS, accompanied by the development of various tools and software tailored for achieving the overarching objective. To the best of our knowledge, current surveys focus on specific domains such as cells, nuclei, or tissues, or they provide summaries of existing tools [3].

Inspired by these premises, this study presents a systematic literature review (SLR) providing a comprehensive survey of the state-of-the-art DL methods for MIS. In this review, the evolution DL techniques tailored to specific segmentation challenges, including cell and nucleus segmentation as well as tissue analysis, is explored. Our objective is to consolidate the knowledge accumulated over the last six years, categorizing contributions, methodologies, datasets, and performance evaluations.

To construct this review, this study examined a total of 113 recent research articles published between January 2018 and June 2024, gathered from a diverse array of four article repositories, i.e., SpringerLink, IEEE Xplore, Science Direct, and PubMed. The subsequent sections provide comprehensive insights into the methodologies employed and their applications through a SLR approach.

The rest of this paper is structured as follows: Section 2 provides a background on prominent approaches proposed for image segmentation. Section 3 delineates the applied research methodology employed for synthesizing studies. Section 4 presents an exposition of the reviewed works addressing the topic of MIS, and ultimately, Section 5 delves into the discussion.

## 2. Background

Image segmentation involves partitioning an image into distinct regions based on specific properties of interest. Traditional segmentation techniques encompass approaches like edge detection, threshold processing, region growing, texture analysis, watershed algorithms, and others.

Nonetheless, each of these techniques comes with its own limitations. DL has emerged as a widely adopted approach for image segmentation across various domains. Image segmentation can be categorized into two main types: semantic-level segmentation and instance-level segmentation. Semantic segmentation classifies each pixel in an image into the foreground and background [3]. Instance-level segmentation is built upon target detection, a process that involves identifying and outlining individual objects within an image, providing unique labels for each instance [3].

In the dynamic field of DL for MIS, several architectural paradigms have emerged as powerful tools. This section spotlights five influential architectures: U-Net, Region-based Convolutional Neural Networks (R-CNN), Generative Adversarial Networks (GANs), You Only Look Once (YOLO), and Vision Transformers (ViTs), which are pivotal in addressing various challenges in image analysis and segmentation.

### 2.1. U-Net

U-Net, introduced by Ronneberger et al. in 2015 [9], stands as a seminal architecture in semantic segmentation, particularly in biomedical image analysis. Its U-shaped design, with skip connections, facilitates detailed feature extraction, making it well-suited for tasks like cell segmentation in microscopic images. Studies employing U-Net have significantly contributed to the precision and efficiency of segmentation outcomes.

**Figure 1:**
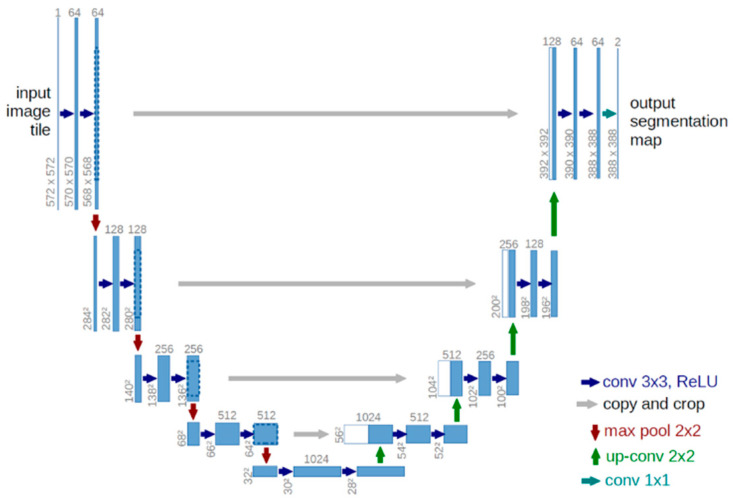
The structure of U-Net [9]. This figure depicts the U-Net structure, highlighting the contracting and expansive paths, with emphasis on the skip connections that facilitate efficient feature integration and detailed pixel-wise segmentation. The model processes an input of 572 × 572 pixels and generates an output of 388 × 388 pixels. Image from [9].

The U-Net architecture consists of a U-shaped channel incorporating skip connections. The encoder comprises four submodules, each housing two convolutional layers, and after each submodule, downsampling is achieved through max pooling. The decoder, with four submodules, progressively increases resolution through upsampling, ultimately providing pixel-wise predictions. Illustrated in Figure 1, the network takes a 572 × 572 input and produces a 388 × 388 output. A distinctive feature is the utilization of skip connections, linking the output of a submodule in the encoder with the input of the corresponding submodule in the decoder, promoting seamless information transfer across network layers. In extending the capabilities of U-Net for more complex tasks, variations such as 3D U-Net and V-Net have emerged, showcasing adaptability to three-dimensional image data.

### 2.2. R-CNN

Introduced by Girshick et al. in 2014 [10], Region-based Convolutional Neural Networks (R-CNNs) have revolutionized the field of object detection and segmentation. Through a two-step process involving region proposal generation and subsequent CNN-based feature extraction, R-CNN and its variants demonstrate exceptional accuracy in localizing objects. Their applications extend to MIS, showcasing their effectiveness.

**Figure 2:**
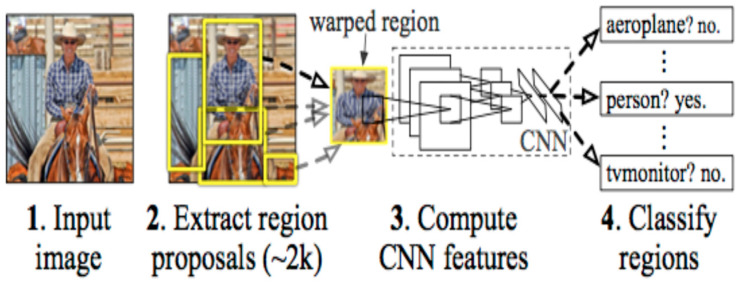
The structure of R-CNNs [10]. This figure outlines the R-CNN architecture, illustrating the process of region proposal generation and CNN-based feature extraction for object detection and classification. Image from [10].

The R-CNNs architecture, as presented in [10], employs a region proposal network to generate bounding boxes using a selective search process. These region proposals undergo warping to standard squares and are then fed into a CNN to produce a feature vector map as the output. The output dense layer comprises features extracted from the image, subsequently utilized by a classification algorithm to classify objects within the region proposal network. Additionally, the algorithm predicts offset values to enhance the precision of the region proposal or bounding box. The sequential processes performed in the R-CNN architecture are visually depicted in Figure 2. Three major variations of the R-CNN model, including Fast R-CNN, Faster R-CNN, and Mask R-CNN, have been introduced in the literature. This progression signifies a refinement in object detection and segmentation methodologies.

### 2.3. GAN

Introduced by Goodfellow et al. in 2014 [11], Generative Adversarial Networks (GANs) have revolutionized the field of image synthesis and generation. Although originally designed for broader applications, GANs have found utility in the augmentation of microscopic image datasets, generating synthetic images for training segmentation models. This unconventional method of data augmentation has attracted interest due to its potential to enhance the robustness of segmentation models.

**Figure 3:**
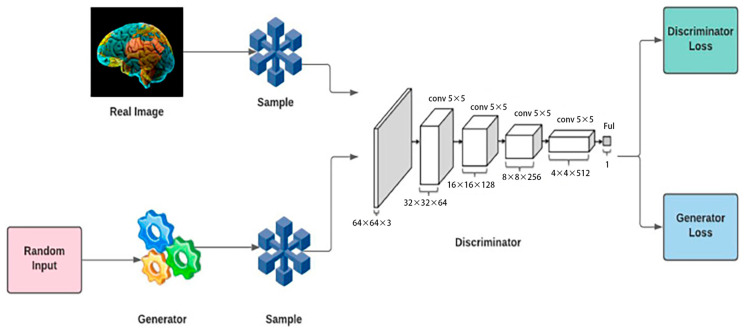
The structure of GANs [11]. This diagram illustrates the structure of a GAN, comprising a generator and a discriminator. The generator, using a random input, produces synthetic images that mimic real images, such as the brain image shown.

Figure 3 shows a block diagram representation of a GAN, which consists of two main components: the generator and the discriminator. The generator, starting with a random input, creates synthetic images that mimic real images, such as the brain image shown. These generated images, along with real images, are fed into the discriminator, a CNN with multiple layers, each reducing in size while increasing the number of filters to analyze features and distinguish between real and fake images. The discriminator calculates a Discriminator Loss based on its ability to correctly classify images, while the generator has a Generator Loss based on its success in “fooling” the discriminator. This adversarial process allows both networks to improve, with the generator producing more realistic images and the discriminator enhancing its ability to differentiate them.

### 2.4. YOLO

YOLO, introduced by Redmon et al. in 2016 [12], has emerged as one of the most efficient architectures for real-time object detection and segmentation tasks. Unlike traditional methods that rely on a region proposal network, YOLO predicts bounding boxes and class probabilities directly from full images in a single pass. This single-shot detection mechanism drastically improves speed while maintaining competitive accuracy, making it particularly useful for real-time applications, including microscopic image segmentation.

YOLO has been adapted for instance-level segmentation tasks, offering efficient detection of individual cells or other biological structures in microscopy images. The most recent versions, YOLOv4 and YOLOv5, have introduced several improvements, such as better feature extraction capabilities using CSPNet and PANet architectures, resulting in enhanced precision for fine-grained segmentation tasks. YOLO’s ability to process entire images quickly and its suitability for applications requiring fast inference make it a promising tool for dynamic fields like medical imaging.

**Figure 4:**
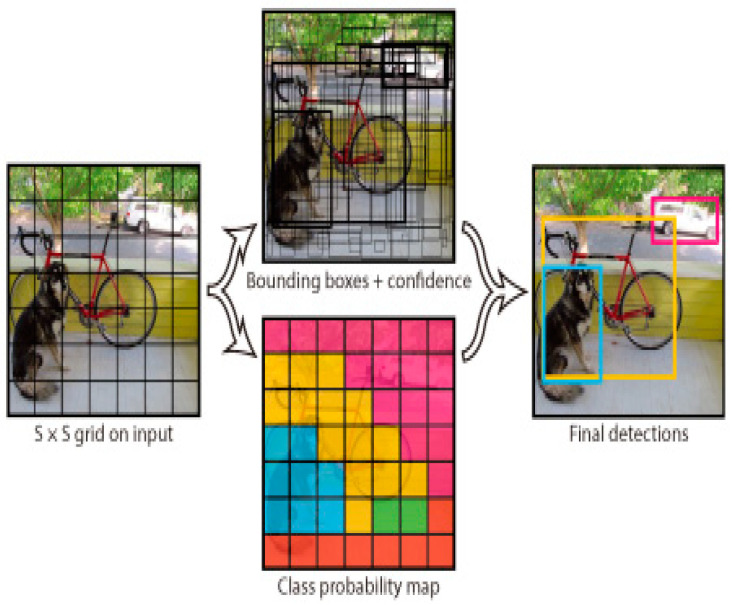
Main idea of YOLO [12]. This figure illustrates the YOLO architecture, showcasing its single-shot detection approach that predicts bounding boxes and class probabilities directly from full images for real-time object detection and segmentation. Image from [12].

Furthermore, YOLO models have been fine-tuned for biomedical tasks, such as detecting nuclei in histopathology images or segmenting tissues in real time during surgery, where both speed and accuracy are critical. Figure 4 illustrates the YOLO architecture for computer vision and its real-time detection pipeline.

### 2.5. ViTs

**Figure 5:**
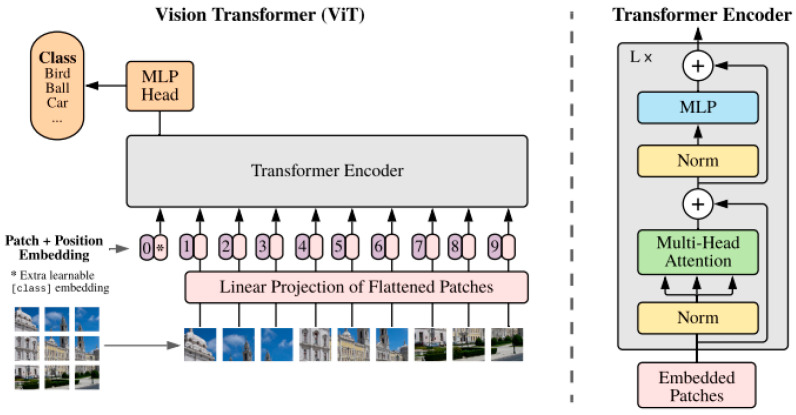
Architecture of the ViT from [13]. This figure illustrates the process of dividing an input image into non-overlapping patches, transforming these patches by adding learnable embeddings, and feeding them through multiple layers of multi-head self-attention and feed-forward networks. Image from [13].

Introduced by Dosovitskiy et al. in 2020 [13], Vision Transformers (ViTs) represent a significant advancement in image analysis by applying transformer architectures to visual data. Unlike CNNs that use localized filters, ViTs treat image patches as tokens, allowing them to capture long-range dependencies and global context. This makes ViTs particularly effective for medical image segmentation, especially in complex scenarios where traditional methods may falter. ViTs can be employed as standalone models or integrated with architectures like U-Net, enhancing accuracy in segmenting intricate structures in medical images. Figure 5 presents the principle of the ViT.

This figure illustrates the process of dividing an input image into non-overlapping patches, transforming these patches by adding learnable embeddings, and passing them through multiple layers of multi-head self-attention and feed-forward networks.

## 3. Methodology

To restrict this article, this study applies the SLR methodology as conducted by Brereton et al. [14]. The primary objective of this approach is to analyze data extracted from the selected studies in the article. The SLR for MIS unfolds through a three-step process:

1. Planning the Review: This step involves specifying the requirements for the review process and forming the questions necessary for the study.
2. Conducting the Review: This step includes finding relevant works and assessing the quality of the research.
3. Documenting the Review: This step involves reporting the selected studies in a paper.

- *Planning the Review*

The research planning phase includes defining research questions. Our SLR aims to address the following research questions:

RQ1: What are the state-of-the-art deep learning techniques for microscopic image segmentation, their primary applications, and the datasets commonly used in the field?

RQ2: How does microscopic image segmentation contribute to the analysis of cells, nuclei, and tissues in biomedical research and medical diagnosis?

RQ3: Are there software tools available for the automated segmentation of microscopic images?

- *Conducting the Review*

We selected articles from a variety of sources based on criteria including study titles, relevant keywords, abstracts, and conclusions. To ensure a full coverage of pertinent literature, we conducted an extensive search across multiple prominent publication databases. These databases include the following:

- IEEEXplore—(https://ieeexplore.ieee.org/) (accessed on 8 October 2024)
- Science Direct—(https://www.sciencedirect.com/) (accessed on 8 October 2024)
- Springer Link—(https://link.springer.com/) (accessed on 8 October 2024)
- PubMed—(https://www.ncbi.nlm.nih.gov/pubmed/) (accessed on 8 October 2024)

This approach ensured that we grouped a diverse and comprehensive selection of articles for our research review. This involved searching through multiple articles by incorporating specific keywords. The searches were conducted across four databases, utilizing the keywords “microscopic image”, “segmentation”, and “deep learning”. We focused on articles published in English between January 2018 and June 2024 to ensure the inclusion of recent research findings.

To establish inclusion and exclusion criteria, this paper adhered to the Preferred Re-porting Items for Systematic Reviews and Meta-Analyses (PRISMA) procedures as outlined by Moher et al. in 2009 [13].

**Figure 6:**
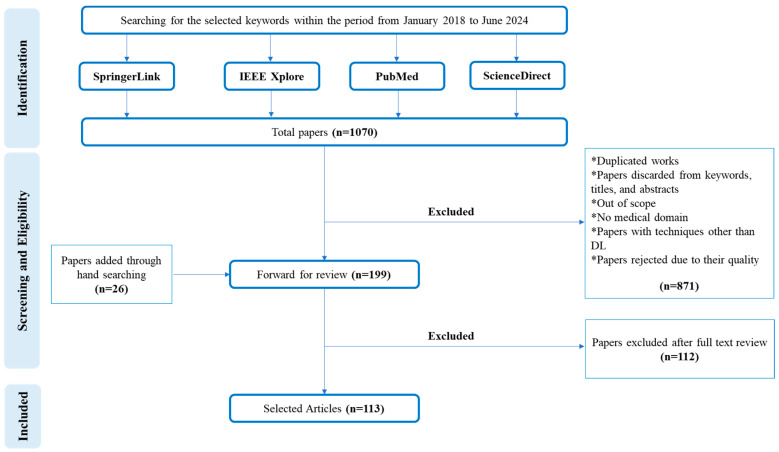
PRISMA flow diagram of the literature selection process. This diagram illustrates the systematic review process undertaken to select relevant articles for the study. It details the total number, the filtering criteria applied, and the final count of articles included in the SLR.

This approach ensured that this study gathered a diverse and comprehensive selection of articles for the research review. Figure 6. illustrates the structured PRISMA framework that guided our process for selecting studies.

The criteria are integrated to ensure that the selected studies align with the specific boundaries and objectives of the research topic. All articles were included based on their relevance to the field of MIS, with a particular focus on DL methods. The selection criteria should be thoughtfully designed to effectively address the research questions, ensuring that they can be clearly interpreted and accurately categorize the pertinent studies.

In conducting this SLR, this study aimed to identify a comprehensive set of relevant studies. However, we encountered limitations in accessing some key publications, particularly those available through IEEE and other subscription-based databases. This lack of access may have resulted in the exclusion of potentially relevant studies, and thus the selection of articles for this review reflects only those for which full texts were accessible.

In accordance with Figure 6, the SLR process was conducted in accordance with the PRISMA guidelines, encompassing three main stages: Identification, Screening, and Inclusion. Initially, a comprehensive search was performed for studies published between January 2018 and June 2024, yielding a total of 1070 papers. This pool was enriched by hand searching, which added 26 additional papers. During the screening phase, 871 papers were excluded for various reasons, including duplication, irrelevance to the medical domain, use of techniques other than DL, or quality issues as determined by the titles, abstracts, and keywords. Following this, 199 papers were forwarded for full-text review. Ultimately, 113 papers were included in the SLR, while 112 papers were excluded after the full-text assessment, ensuring a focused and relevant collection of studies for analysis.

In addition, to extend the search and find more relevant literature, we also performed a hand-searching process where we combined several keywords related to the underlying research.

Following this process, the remaining texts were subjected to an in-depth reading to achieve a more comprehensive understanding. In this final stage, a careful analysis was performed to validate the relevance of each study, ultimately leading to the examination of 113 articles.

In Section 4, this study goes a step further in documenting the review to address responses to the research questions.

## 4. Analysis of the Papers

In this section, this study provides an analysis of the papers included in the review. Section 4.1 will focus on papers that address RQ1 and RQ2, while Section 4.2 will cover papers addressing RQ3.

Table 1 contains a list of abbreviations used in this section.

### 4.1. RQ1 and RQ2

In this section, we address two key research questions to comprehensively explore the landscape of MIS in the context of DL techniques and their applications in biology research.

RQ1: What are the state-of-the-art deep learning techniques for microscopic image segmentation, their primary applications, and the datasets commonly used in the field?

RQ2: How does microscopic image segmentation contribute to the analysis of cells, nuclei, and tissues in biology research?

**Figure 7:**
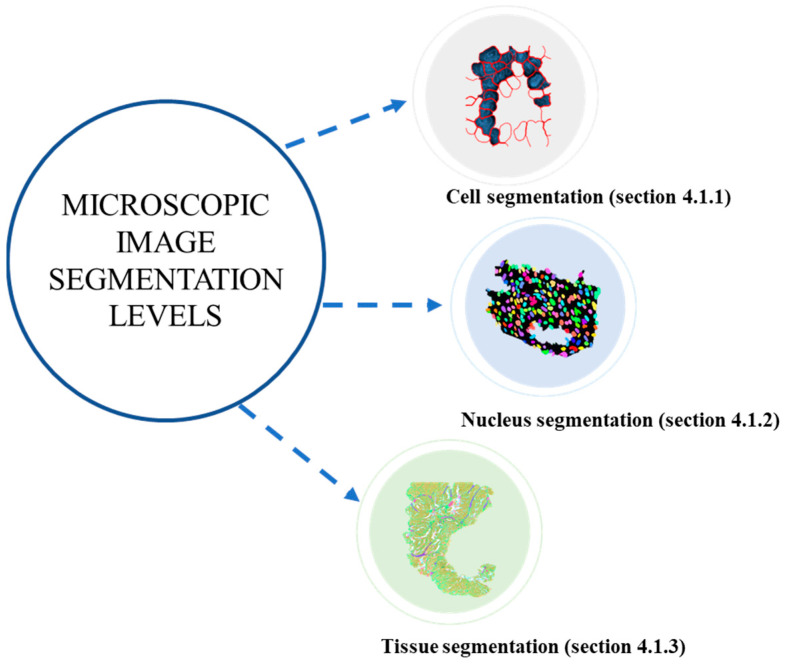
Microscopic image segmentation levels. This figure illustrates the segmentation process across three levels: cell, nucleus, and tissue.

In the context of MIS, there are three distinctive levels of analysis (see Figure 7), each tailored to address specific research and diagnosis needs.

The first level is cell segmentation, which involves the precise delineation and categorization of individual cells within an image. This allows researchers to study cell morphology, spatial distribution, and behavior under various conditions.

Moving deeper, nucleus segmentation represents the second level. Here, the focus is on accurately identifying and segmenting the cell nucleus within each cell. This level of segmentation is pivotal in understanding genetic and cellular processes, as the nucleus houses the cell’s genetic material.

The third level, tissue segmentation, extends the analysis to a broader scale. It entails the partitioning of an image into different tissue types or regions, providing insights into the composition and structure of the tissue sample. Tissue segmentation is vital in applications like disease diagnosis, histology, and pathology, enabling the identification of various tissue components, such as epithelial and connective tissues, blood vessels, and tumors.

These three levels of MIS, cell, nucleus, and tissue segmentation, collectively contribute to a comprehensive understanding of biological structures and processes, catering to diverse research objectives in fields such as biology and pathology.

To cover these three levels, this study provides an overview of various research findings specific to each level, as detailed in the sections for cell segmentation, nucleus segmentation, and tissue segmentation, respectively.

#### 4.1.1. Cell Segmentation

Table 2 provides an overview of selected studies focused on cell segmentation, reflecting the diversity of methodologies and applications in this domain. As depicted in the table, distinct studies present diverse approaches tailored for specific tasks, primarily emphasizing either semantic or instance segmentation, with some studies adopting a hybrid approach incorporating both techniques.

In this section, we will investigate into an examination of various studies, categorizing them based on their approaches to semantic and instance segmentation.

Starting with a focus on semantic segmentation, in their publication [15], the authors introduced a method derived from the GAN approach. One notable strong point of this method is its avoidance of the need for formulating a loss function during the optimization process. This approach demonstrates promising segmentation results on real fluorescent microscopy data called H1299 dataset [16].

Additionally, in [17], the authors introduced a workflow employing DNN for cell segmentation specifically applied to PCI. The proposed pipeline involves three stages: the first stage focuses on formulating PCI, the second stage utilizes DNN for image restoration, and the third stage highlights the advantages of artifact-free images for segmentation. The evaluation was conducted on an adapted dataset of phase-contrast microscopy image sequences. The results demonstrated favorable outcomes compared to some SOTA approaches in terms of ACC, achieving a value of 0.908, IoU with a score of 0.4698, and DSC, attaining a value of 0.6859.

The method combining Automatic Enhancement Preprocessing (AEP) [18] and Automatic Weighted Ensemble Learning (AWEL) significantly improves cell image segmentation, as demonstrated by both qualitative and quantitative results. The combined AEP–AWEL approach quantitatively enhances IoU scores by approximately 2.55% for human iRPE cell images, 1.41% for cell nuclei, and 2.95% for mouse liver cell membranes compared to the standard U-Net without preprocessing, resulting in an overall average IoU improvement of 1.63%. Additionally, the DSC was improved by about 2.24% for iRPE cells, 1.04% for cell nuclei, and 3.00% for mouse liver cell membranes. Qualitatively, AEP enhances visual clarity, making previously indistinct nuclei and membranes clearer, especially in low-quality images, leading to superior segmentation results compared to traditional methods like U-Net. The integration of AEP and AWEL notably enhances segmentation accuracy and visual quality.

In another study, the authors in [19] introduced the CellSegUNet model, which combines the architectures of UNet++ and Residual U-Net to form a robust framework for cell segmentation, successfully tackling challenges like varying image intensities, overlapping nuclei, and noise. By incorporating an attention mechanism and optimized residual connections, the model enhances feature representation and captures intricate details in complex images. It underwent thorough evaluation across several prominent datasets, yielding remarkable results: an ACC of 0.980, DSC of 0.970, and JI of 0.959 on the Data Science Bowl (DSB); an ACC of 0.931, DSC of 0.957, and JI of 0.829 on the Sartorius Cell Instance Segmentation (SCIS); and an ACC of 0.976, DSC of 0.971, and JI of 0.927 on Blood Cell Segmentation (BCS). Comparisons demonstrate that CellSegUNet outperforms both UNet++ and ResUNet models of similar depth. Overall, its innovative attention mechanisms, enhanced feature representation, and lightweight design make CellSegUNet an effective tool for real-world cell segmentation applications.

The Lite-UNet [20] model offers an efficient solution for cell localization in pathological image analysis, addressing challenges such as large color variations, uneven cell distribution, and overlapping borders. It integrates three key modules: the Gradient Aggregation (GA) module, which enhances gradient information to counteract color disparities; the Ghost_CBAM module, which reduces computational costs while handling uneven cell distributions; and the Graph Correlation Attention (GCA) module, which captures higher-order feature associations for better localization. Lite-UNet outperformed traditional models across several datasets, achieving an MAE of 18.1 and an F1 of 0.765 on the BCData dataset. Additionally, it maintained a strong performance on other datasets while reducing model parameters to just 3.7% of U-Net and computational complexity to 6.7%, making it a promising lightweight solution for accurate cell localization.

The TransGuider method proposed in [21] presents an innovative approach to medical image segmentation by integrating an external–latent attention mechanism. Its key components include a Latent Attention Module, which utilizes an enhanced entropy quantification method to effectively locate latent contextual information, and an External Self-Attention Module, which leverages sparse representation to preserve global contextual details while minimizing computational costs. Additionally, the Multi-Attention Collaborative Module further refines segmentation accuracy by guiding the network to focus on regions of interest. TransGuider demonstrated superior performance, achieving an MDSC of 0.9516 and MIoU of 0.9091 on the CVC-ClinicDB dataset, and improving results on the COVID-19CT100 dataset. Although its performance on certain datasets, such as the 2018 DSB and ISIC 2018, was slightly lower than other methods, TransGuider remains a competitive and effective solution for medical image segmentation.

The authors in [22] introduce the Fourier Adaptive Recognition System (FARS), designed to improve medical image analysis for detecting malarial parasites. By shifting from bounding box annotations to semantic segmentation labels, FARS enables more detailed examination of microscopy slides. It employs adversarial training and Color Domain Aware Fourier Domain Adaptation (F2DA) for robust feature extraction across various microscopy types and magnifications. FARS features a specialized encoder–decoder architecture optimized for malaria detection, supported by multiple loss functions and a Category-Dependent Context Attention module that highlights relevant features. The system demonstrates significant performance improvements, with cross-magnification MAP increasing from 0.313 to 0.551, and a 15.68% boost in cross-domain adaptability. Overall, FARS effectively delivers reliable predictions in medical image analysis across diverse microscopy settings.

Saliency Guided Attention Module Network (SGAM-net) proposed in [23], introduces a novel approach to cell boundary segmentation by combining traditional image features with DL techniques. Unlike conventional models, SGAM-net frames cell segmentation as a boundary prediction task, improving the separation of overlapping cells. Through a specialized attention mechanism, the model effectively integrates perceptual features, enhancing segmentation quality while requiring less annotated data. SGAM-net’s minimal complexity allows for efficient training on smaller datasets, addressing challenges in data-limited scenarios. In evaluations across three public microscopy datasets, SGAM-net demonstrated substantial improvements in performance, with DSC and IoU scores surpassing the baseline U-Net model by 1.36% to 6.35% and 2.31% to 6.99%, respectively. Additionally, it required 23.07% fewer parameters than the UNet++ model. In cell counting tasks, SGAM-net outperformed the state-of-the-art StarDist model, achieving lower mean absolute errors. These results highlight SGAM-net’s efficiency and effectiveness in addressing complex cell segmentation challenges.

In [24], the authors introduce an efficient algorithm for segmenting and classifying HeLa cells by shape and life cycle stage, utilizing a hybrid method that integrates multiple CNN architectures within the U-Net framework. High-resolution time-lapse images were obtained with a specialized microscope to reduce optical distortions. Among several U-Net variants tested, including Simple U-Net, VGG19-U-Net, Inception-U-Net, and ResNet34-U-Net, the ResNet34-U-Net proved most effective, achieving an IoU of 0.8067, a DSC of 0.8873, and an ACC of 0.9909. Although Inception-U-Net and VGG19-U-Net showed slightly lower performance, and Simple U-Net performed the least effectively, ResNet34-U-Net demonstrated robustness in handling overlapping or indistinct cells. These results emphasize the strong potential of hybrid U-Net models, particularly ResNet34-U-Net, for HeLa cell segmentation, with promising applications for other cell lines and automated biomedical image analysis.

In [25], the authors introduced an improved U-Net algorithm named McbUnet, which incorporates mixed convolution blocks, combining the advantages of U-Net and residual learning. The effectiveness of this proposed approach was validated using the 2018 DSB. McbUnet demonstrated outstanding performance compared to standard U-Net, MultiResUNet [26], and CE-Net [27]. Notably, the method achieved notable results in terms of ACC with a value of 0.956 and IoU with a value of 0.816.

In [28], DOLG-NeXt was introduced in the context of cell contour segmentation. DOLG-NeXt incorporates SE-Net-driven ConvUNeXt architecture, coupled with multi-scale feature aggregation through the DOLG module. This is followed by the inclusion of a channel attention mechanism, aiming to capture high-level feature representations that preserve both spatial and channel information. DOLG-NeXt demonstrated superior performance compared to other SOTA architectures, including U-Net and Transformer-based variants, across four benchmark public datasets representing electron microscopy, colonoscopy, fluorescence, and retinal modalities. On the ISBI 2012 [29], CVC-ClinicDB 2018 [30], 2018 DSB [31], and DRIVE datasets, DOLG-NeXt achieved remarkable DSC values of 0.958, 0.951, 0.947, and 0.848, along with MIoU scores of 0.901, 0.918, 0.889, and 0.735, respectively.

In [32], a different application, which centers on semantic cell segmentation, was introduced as GRUU-Net. This framework integrates the iterative refinement of feature maps through GRU with multi-scale feature aggregation using a U-Net. For more enhancement of training robustness and segmentation performance, the authors introduced a novel normalized focal loss designed for a momentum-based optimizer. Despite being characterized by a reduced number of parameters, the proposed network achieved superior or competitive results across the majority of the used datasets. Notably, the authors trained the network using only a few example images and did not employ hand-crafted weighting of the cross-entropy loss. For instance, in the case of the glioblastoma dataset [33]. GRUU-Net demonstrated a favorable DSC of 0.933, surpassing the performance of both U-Net and ASPPU-Net [34].

In [35], the authors presented SBU-net, aiming to improve segmentation performance by incorporating perceptual features such as saliency and ballness. This innovative approach yields superior results when applied to bright-field microscopic images. In-depth insights into its effectiveness were gained through a comprehensive evaluation, comparing SBU-net with established models including U-Net, U-Net++ [36], Link-net [37], and Attention U-Net. Experimental results highlight the exceptional performance of SBU-net, demonstrated by significant enhancements in both IoU and DSC metrics compared to SOTA models. It attained an MIoU of 0.804 and 0.829, along with an MDSC of 0.891 and 0.906, respectively, on two publicly available bright-field datasets of T cells and pancreatic cancer cells. To evaluate the model’s ability to generalize across different microscopy types, the authors conducted tests on a fluorescence dataset.

Additionally, the incorporation of pre-trained networks proves advantageous in the case of semantic cell segmentation. In their work [38], the authors proposed Aura-net, which integrates a pre-trained ResNet-18 with an Attention U-Net and undergoes training utilizing an AC loss. The authors conducted experiments on three publicly available PC microscopy image datasets. The results showcased that Aura-net outperformed SOTA approaches such as the standard U-Net, CE-Net, and Attention U-Net [39]. The proposed method achieved a DSC of 0.846 for the first database, 0.877 for the second database, and 0.818 for the third database.

As presented in Table 2, some studies have suggested various approaches for cell (nuclei) segmentation, indicating the segmentation process for both entire cells and their respective nuclei. In this context, starting with the study outlined in [40], the authors introduced AS-UNet tailored for this segmentation task. This framework consists of three parts: encoder module, decoder module, and atrous convolution module. Their experimentation focused on two datasets, namely the MOD dataset [41] and the BNS dataset [42]. Comparative analyses were conducted, pitting the AS-UNet method against other published SOTA models, such as PSPNet [43], ENET [44], SegNet [45], and Link-net. The outcomes underscored the superiority of the AS-UNet algorithm, particularly excelling in scenarios involving multi-cell adhesions and small-sized cells. Particularly on the MOD, it achieved an ACC of 0.928. Moreover, when assessed on the BNS dataset, the AS-UNet algorithm achieved an ACC of 0.968.

In the same context, a feedback attention network called FANet [46] was proposed. It utilizes information from each training epoch to refine the prediction maps in subsequent epochs, allowing the architecture to self-rectify predicted masks. This self-correction mechanism contributes to accurate and consistent segmentation results across diverse datasets. The performance of FANet is comprehensively evaluated against SOTA DL methods on seven publicly available biomedical imaging datasets.

In the cited reference [47], the authors introduced UNet++, an algorithm presenting a deeply-supervised encoder–decoder network architecture tailored for medical image segmentation. This architecture establishes connections between the encoder and decoder sub-networks through nested, dense skip pathways, aimed at minimizing the semantic gap in their feature maps. The performance of UNet++ was assessed in contrast to U-Net and wide U-Net architectures across various medical image segmentation tasks. These tasks encompassed nodule segmentation in low-dose CT scans of the chest, nuclei segmentation in microscopy images, liver segmentation in abdominal CT scans, and polyp segmentation in colonoscopy videos. The paper extensively compared the performance of UNet++ with U-Net and wide U-Net, highlighting the superior segmentation results achieved by UNet++.

The SEAM-Unet++ [48] model, specifically designed for cell segmentation, underwent rigorous comparisons with several established models, including UNet++, U-Net, PSPNet, DeepLab, and Kun Lan’s model. While PSPNet emphasizes multi-scale contextual information and DeepLab enhances object boundary detection through techniques like spatial pyramid pooling, both Kun Lan’s model and SEAM-Unet++ build on the UNet++ framework for improved cell segmentation. SEAM-Unet++ outperformed the others, achieving the lowest ACC loss of 0.1037 and an impressive IoU score of 0.9101, demonstrating its precision in identifying target regions. The model also excelled in Re, Pre, and DSC metrics, proving its reliability for real-world applications, particularly in segmenting overlapping cell contours. Its innovative integration of the SEAM attention mechanism with UNet++ effectively addressed interclass occlusion and enhanced feature representation, positioning it as an optimal choice for FISH image segmentation.

The study introduces the Microbiological Rapid On-Site Evaluation (M-ROSE) system [49], a DL-based approach for automatic pathogen identification in clinical specimens. Using 1705 high-resolution Gram-stained images from patients with lung infections, the system identified 4833 cocci and 6991 bacilli, carefully labeled and categorized. YOLOv5 was employed for object detection to classify cocci and bacilli, while U-Net enabled detailed bacterial segmentation. Benchmark results showed significant improvements in identification speed and accuracy over traditional methods, suggesting that M-ROSE could enhance diagnostic precision and treatment effectiveness for pulmonary infections. Additionally, the dataset generated offers a valuable resource for further research on automated bacterial identification, addressing the urgent challenges of hospital-acquired infections and multidrug resistance.

The study evaluates the csPWS-seg [50] model for nuclei segmentation using HCT116 live-cell imaging data with four distinct training approaches based on different csPWS feature images: bright-field (BF), Σ, and spatial wave (SW). The SW-only model achieved the best results, with a median IoU of 0.78 and a DSC of 0.86. In comparison, models trained on BF and Σ images had lower scores due to their dependence on dataset-specific features. A combined model trained on all feature images showed further improvement, reaching a median IoU of 0.80 and a DSC score of 0.88, highlighting the value of diverse imaging data for enhancing segmentation accuracy in csPWS microscopy.

Now, with regard to instance segmentation. In [51], authors proposed GeneSegNet. It employs a recursive training strategy to handle noisy training labels. In this study, GeneSegNet’s performance was systematically assessed by benchmarking it against five alternative methods: the watershed algorithm, Cellpose [52], JSTA [53], Baysor [54] and Baysor(prior) [54]. The comparative analysis revealed that GeneSegNet outperforms existing methods in cell segmentation by effectively leveraging gene expression information and optimizing the use of imaging data. GeneSegNet produces more accurate cell boundaries, encompassing a greater number of RNA reads within cells, while mitigating the issue of oversegmentation.

Sukumarran et al. [55] present an improved approach for detecting malaria-infected red blood cells (RBCs) using a modified YOLOv4 model. Their method includes three main steps: training the original YOLOv4 as a baseline, applying layer pruning to the C3, C4, and C5 Resblock bodies to reduce overfitting, and replacing the CSPDarknet53 backbone with a lighter ResNet-50 backbone to lower computational complexity. Testing on two datasets showed that the pruned YOLOv4-RC3_4 model achieved a 0.907 detection ACC, surpassing the original model and reducing computational load by 22% and model size by 23 MB. These enhancements demonstrate the model’s potential for improved accuracy and efficiency in malaria-infected cells.

The study in [56] demonstrates notable advancements in cell segmentation, especially in scenarios with limited annotated data. By employing a CycleGAN framework, the proposed method achieved a DSC of 0.853, a substantial improvement from the 0.725 DSC observed in baseline models that did not utilize synthetic data. The IoU metrics further confirmed this enhancement in segmentation accuracy. Qualitatively, the synthetic images generated by the CycleGAN expanded the training dataset’s size and diversity, facilitating better generalization across various cell types and appearances. This enhanced data distribution minimized overfitting, resulting in lower validation losses and a more stable training process. The model proved robust in challenging scenarios, effectively addressing variations in cell morphology and staining characteristics. These findings highlight the effectiveness of CycleGANs in augmenting limited datasets and their potential to advance medical image analysis in cell segmentation tasks.

DaCSeg, proposed in [57], employs a specialized DL framework to improve feature extraction and segmentation accuracy, particularly for overlapping chromosomes. Its architecture integrates a backbone network for robust feature extraction alongside optimized convolutional layers that effectively capture both local and global contextual information. A distinctive mechanism leverages shape prior knowledge to accurately differentiate between various chromosome types and configurations. Evaluation results reveal that DaCSeg achieves a MAP of 0.841, outperforming traditional methods and demonstrating a 4.68% improvement over Mask R-CNN. Visualization analyses further confirm DaCSeg’s strong segmentation capabilities in challenging scenarios, consistently identifying overlapping chromosomes while minimizing mask loss. Overall, DaCSeg proves to be a highly effective tool for complex chromosome segmentation, marking a significant advancement in precision within digital pathology.

The LKCell [58] method introduces a novel approach to cell nuclei instance segmentation, using large convolution kernels to expand the receptive field and improve segmentation without the need for stacking smaller kernels. This design enhances performance while reducing computational demands, achieving a 78.4% reduction in FLOPs compared to previous state-of-the-art methods. LKCell sets new benchmarks on the PanNuke dataset, with a mPQ score of 0.508 and a bPQ score of 0.684. Its effectiveness in segmenting complex cellular structures and overlapping nuclei, combined with its efficient parameter usage, marks LKCell as a major advancement in automated cell segmentation for medical applications.

The proposed method [59] enhances segmentation and counting of senescent mesenchymal stem cells (MSCs) in bright-field microscopy images through a DL approach utilizing Mask R-CNN and self-supervised learning (SSL). By incorporating contrastive learning in SSL, the model effectively leverages unlabeled data, leading to significant performance improvements. With a ResNet-101 and Feature Pyramid Network (FPN) backbone pre-trained on MSCOCO, and further optimized through SSL using SimCLR, the model achieved an 8.3% increase in MAP at IoU 0.5 and a 4.2% boost for IoU between 0.5 and 0.95. It also demonstrated higher Pre, Re, and DSC, outperforming models like U-Net and DeepLabV3, making it a robust solution for automated senescent cell quantification in clinical settings.

The CellViT-SAM-H [60] model offers an advanced approach for nuclei detection and segmentation in histopathological images by integrating a Vision Transformer (ViT) architecture with a segmentation attention mechanism (SAM) to capture local and global features effectively. Enhanced by extensive data augmentation and a custom loss function combining focal and Dice loss to tackle class imbalance, CellViT-SAM-H achieves notable improvements. On the PanNuke dataset, it attained a Pre of 0.85, Re of 0.83, and F1 of 0.84 in nuclei detection, marking a 26% increase in F1, Epi-score for epithelial nuclei detection. For segmentation tasks, the model recorded a PQ of 0.75 across 19 tissue types, peaking at 0.79, surpassing other models like Hover-Net (0.71) and StarDist (0.66). These results underscore CellViT-SAM-H’s significant advantages in detection and segmentation accuracy.

In [61], a robust framework for cell instance segmentation was introduced. This framework, built upon Mask R-CNN, is designed to generate cell segmentation without the need for additional postprocessing steps. To enhance the model’s capability in learning segmentation boundaries, the authors incorporated the use of Shape-Aware Loss, a distance-based pixel-wise weighted cross-entropy loss. The proposed framework exhibits a strong performance, surpassing other models mentioned in the paper, achieving IoU values of 0.919 and 0.949 for the DICC2DH-HeLa and PhCC2DH-U373 datasets, respectively.

In [62], an approach integrates convolutional LSTM with the U-Net architecture used for instance cell segmentation and tracking in time-lapse microscopy. The integration of spatio-temporal considerations in this method enhances its capability to accurately delineate and track individual cells over consecutive frames in time-lapse microscopy sequences. The method’s performance was evaluated using the Cell Tracking Challenge, resulting in SOTA outcomes. It achieved the top position for the Fluo-N2DH-SIM+ dataset and the second position for the DIC-C2DLHeLa datasets.

Authors in [63] proposed a method, attentive instance segmentation, that combines a single-shot multi-box detector (SSD) and a U-Net. It employs attention mechanisms in both the detection and segmentation modules to focus on useful features. Quantitative and qualitative results show that the proposed approach achieves higher ACC and faster speed compared to the SOTA methods.

The study outlined in [64] introduces Cell T-Net. The efficacy of this approach was assessed using the LIVECell and Sartorius datasets. To demonstrate the prowess of Cell T-Net, the authors replicated several SOTA object detection and segmentation models using the Detectron framework. Specifically, they recreated four one-stage models—SSD [65], RefineDet [66], RetinaNet [67], and CornerNet [68]—as well as three two-stage models, including Faster R-CNN, Mask R-CNN, and Cascade Mask R-CNN [69]. The results indicate that Cell T-Net surpasses SOTA models, particularly in addressing challenges inherent in the characteristics of cell datasets.

In the study [70], a DL model, leveraging cGANs, was introduced for instance cell segmentation. This approach involves creating synthetic masks through a GAN, specifically StyleGAN2-ada, and generating corresponding synthetic microscopy images using image-to-image translation (pix2pix). This method explicitly generates labeled masks, providing versatility for use in various tasks beyond instance segmentation.

Authors in [71] introduced an algorithm that integrates DL with thresholding and watershed-based segmentation. This strategy resulted in an 86% similarity to the ground truth segmentation in the identification and separation of cells and a good average ACC (0.84). However, the algorithm exhibited varying performance levels across different datasets, especially in cases where lower segmentation quality was observed due to increased variability in cell shape and appearance.

The paper referenced in [72] introduces a box-based cell instance segmentation method that integrates keypoint detection with individual cell segmentation. The framework consists of two main branches: a keypoint detection branch and an individual cell segmentation branch, employing a ResNet-50 Conv1-4 as the backbone network. The method identifies five pre-defined points of a cell through keypoint detection, and these points are then organized using a keypoint graph to derive the bounding box for each cell. Within these bounding boxes, cell segmentation is executed on the feature maps. The effectiveness of the proposed method is validated on two cell datasets exhibiting distinct object shapes, showcasing its superior performance compared to other instance segmentation techniques. Qualitative results further affirm the efficacy of the proposed method.

Next, we will introduce the selected studies that have proposed various architectures for both semantic and instance segmentation. Furthermore, the study explored various U-Net architectures [73], including Attention and Residual Attention U-Net, to identify the most suitable architecture for living cell segmentation. The dataset used in this research comprises bright-field transmitted light microscopy images of HeLa cells acquired from different time-lapse experiments. The Residual Attention U-Net demonstrated the best performance, achieving an MIoU of 0.953 and an MDC of 0.975.

In [74], the authors introduced the 3D CellSeg framework designed for both semantic and instance cell segmentation. Experiments on cell segmentation were conducted across four distinct cell datasets. The results demonstrate that 3D CellSeg surpasses the baseline models on the ATAS [75], HMS, and LRP [76] datasets, achieving overall accuracies of 95.6%, 76.4%, and 74.7%, respectively. Additionally, the framework achieves an ACC value comparable to baselines on the Ovules dataset [77], achieving an overall ACC of 82.2%.

Additionally, the CS-Net network [78] was applied. Comparative results with leading lightweight models reveal that the proposed model achieves a more favorable balance between segmentation performance and computational complexity.

Moreover, it is crucial to underscore pertinent research studies that have employed segmentation techniques for cell counting. Cell counting, defined as the process of determining the number of cells within an image or a designated region of interest, plays a pivotal role in various scientific investigations. For instance, in the work in [79], the authors introduced MSCA-UNet, a cell segmentation method specifically tailored for the application of automatic cell counting, utilizing density regression. To demonstrate the effectiveness of their algorithm, the authors conducted a thorough comparison with SOTA methods. The evaluation encompassed three datasets: VGG CELL [80], MBM CELL [81], and ADI CELL [82]. In preparing their inputs for the fully convolutional network, the authors employed preprocessing techniques, including resizing and patch division. These measures were crucial when dealing with input images of varying dimensions and cell densities, especially in the context of extremely high-resolution images featuring high cell densities. Data augmentation was additionally applied to enhance the model’s robustness to different cell orientations. For the evaluation, MAE was employed as a metric for cell tracking. Notably, the proposed network demonstrated the lowest MAE values (2.4, 8.0, 11.5) for the VGG, MBM, and ADI datasets, respectively. In terms of cell detection, the proposed method achieved commendable Pre, Re, and F1 values.

Ref. [83] introduces SAU-Net as an innovative approach for cell counting, specifically designed for application in both 2D and 3D microscopy images. The network extends the U-Net architecture by incorporating a Self-Attention module and integrating Batch Normalization after every convolution and deconvolution layer in U-Net. SAU-Net’s versatility in handling both 2D and 3D images is a notable enhancement. The evaluation of SAU-Net encompassed five public datasets: VGG, MBM, ADI, DCC, and MBC. The proposed method demonstrated impressive Pre values (99.94, 88.76, 88.57, 99.52, and 92.52) for VGG, MBM, ADI, DCC, and MBC datasets, respectively.

In [84], an application of cell counting was explored via concatenated fully convolutional regression network. Experimental studies conducted on four datasets, including synthetic bacterial cells [85], bone marrow cells [80], colorectal cancer cells [86], and human embryonic stem cells [82], highlight the superior performance of the proposed method.

**Figure 8:**
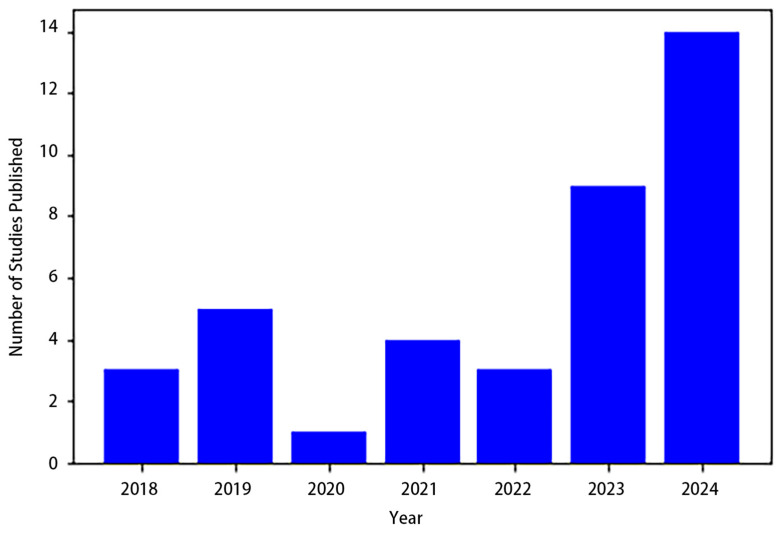
Number of studies published by year for cell segmentation.

In summary, this section explored various studies on cell segmentation. The bar plot (Figure 8) highlights the yearly distribution of studies published on this topic from 2018 to 2024. The data reveal a fluctuating trend, with a notable rise in publications in recent years. Cell segmentation research can be broadly classified into two categories: semantic cell segmentation and instance cell segmentation. In the context of semantic segmentation, AS-UNet stands out, excelling in scenarios with multi-cell adhesions and small-sized cells, achieving ACCs of 0.968 and 0.928 on BNS and MOD datasets, respectively. GAN-inspired methods presented a novel approach, avoiding the need for a formulated loss function during optimization. MSCA-UNet specifically targeted automatic cell counting, demonstrating effectiveness across various datasets. A DNN workflow applied to Phase Contrast Imaging showcased favorable outcomes in ACC, IoU, and DSC. SAU-Net introduced an innovative approach for cell counting in both 2D and 3D microscopy images, exhibiting versatility across different datasets.

**Table 2:** Summary of Cell Segmentation Studies in the Literature. ✓ indicates code availability, ⨯ indicates code not available.

Reference	Publication Year	Method	Task	Dataset	Instance/Semantic /Both	Code Availability	Results
[15]	2018	GAN	Cell segmentation	H1299 data set	Semantic	✓	Pre: 0.899 Re: 0.82 JI: 0.806
[17]	2023	DNN	Cell segmentation	Phase contrast microscopy image sequence	Semantic	⨯	ACC: 0.908 IoU: 0.4698 DSC: 0.6859
[18]	2024	AWEL	Cell segmentation	Mouse Liver Cell Images, Human iRPE Cell Images, ISBI2012 Challenge	Semantic	✓	IoU: Cytoplasm: 0.7191 ± 0.0391 Membrane: 0.4327 ± 0.0232 Nucleus: 0.6342 ± 0.0338 DSC: Cytoplasm: 0.8360 ± 0.0264 Membrane: 0.6036 ± 0.0226 Nucleus: 0.7756 ± 0.0250
[19]	2024	CellSegUNet	Cell segmentation	DSB, Sartorius Cell Instance Segmentation, Blood Cell Segmentation	Semantic	⨯	DSB: ACC: 0.980 DSC: 0.959 JI: 0.927 Sartorius Cell Instance Segmentation: ACC: 0.931 DSC: 0.957 JI: 0.829 Blood Cell Segmentation: ACC: 0.976 DSC: 0.971 JI: 0.927
[20]	2023	Lite-UNet	Cell localization and counting	BCData, Seg_Data, PSU	Semantic	✓	Seg_Data: Counting: MAE: 5.2 Localization: F1/Pre/Re: 0.844/0.858/0.831 PSU dataset: Counting MAE: 33.7 Localization: F1: 0.602 BCData: Counting: MAE: 18.1 Localization: F1/Pre/Re: 0.765/0.770/0.761
[21]	2023	TransGuider	Cell (nuclei) segmentation	MoNuSeg, 2018 DSB, ISIC 2018, CVC–ClinicDB, COVID-19-CT100	Semantic	✓	CVC Clinic DB: MDSC: 0.9516 MIoU: 0.901 COVID-19-CT100: MDSC: 0.7695 MIoU: 0.6488 2018 DSB: MDSC: 0.9242 MIoU: 0.8680 ISIC 2018 MDSC: 0.8948 MIoU: 0.8268
[22]	2024	FARS	Cell segmentation	M5 dataset, mages captured using both LCM and HCM	Semantic	✓	MAP: from 31.3% to 55.19%
[23]	2024	SGAM-net	Cell boundary segmentation	Dataset 1: 57 bright-field images of pancreatic cancer cells, Dataset 2: 209 bright-field images of mouse CD4+ T cells, Dataset 3: 72 fluorescent images of breast cancer cells	Semantic	⨯	Dataset 1: DSC: 0.897 ± 0.009 IoU: 0.815 ± 0.015 Dataset 2: DSC: 0.858 ± 0.013 IoU: 0.752 ± 0.021 Dataset 3: DSC: 0.72 ± 0.11 IoU: 0.581 ± 0.13.
[24]	2024	Hybrid U-Net-based model	Segmenting living HeLa cells and classifying them based on their shapes and life cycle stages	650 images of HeLa cells captured through telecentric bright-field reflected light microscopy	Semantic	⨯	IoU: 0.806 DSC: 0.887 ACC: 0.990
[25]	2020	McbUnet	Cell segmentation	2018 DSB dataset	Semantic	⨯	ACC: 0.956 IoU: 0.816
[28]	2023	DOLG-NeXt	Cell contour segmentation	DRIVE CVC-ClinicDB 2018 DSB ISBI 2012	Semantic	⨯	ISBI 2012: DSC: 0.958 CVC-ClinicDB 2018: DSC: 0.951 2018 DSB DSC: 0.947 DRIVE: DSC: 0.951 ISBI 2012: MIoU: 0.901 CVC-ClinicDB 2018: IoU: 0.918 2018 DSB: MIoU: 0.889 DRIVE: IoU: 0.735
[32]	2019	GRUU-Net	Cell segmentation	DIC-C2DH-HeLa Fluo-C2DL-MSC Fluo-N2DH-GOWT1 Fluo-N2DH-HeLa PhC-C2DH-U373 PhC-C2DL-PSC	Semantic	⨯	DSC: 0.933
[35]	2023	SBU-net	Cell segmentation	Bright-field datasets of T cells Pancreatic cancer cells MCF10DCIS.com cells labeled with Sir-DNA	Semantic	⨯	Bright-field datasets of T cells: IoU: 0.804 DSC: 0.891 Pancreatic cancer cells IoU: 0.829 DSC: 0.906
[38]	2021	Aura-net	Cell segmentation	Microscopy image datasets from the Boston University Biomedical Image Library	Semantic	✓	Dataset 1: DSC: 0.846 IoU: 0.746 Pre: 0.881 Dataset 2: DSC: 0.877 IoU: 0.788 Pre: 0.869 Dataset 3: DSC: 0.818 IoU: 0.697 Pre: 0.730
[40]	2019	AS-UNet	Cell (nuclei) segmentation	MOD dataset BNS dataset	Semantic	⨯	MOD dataset: ACC: 0.928 BNS dataset: ACC: 0.968
[46]	2022	FANet	Cell (nuclei) segmentation	Kvasir-SEG CVC-ClinicDB Dataset: 2018 DSB ISIC 2018 Dataset DRIVE Dataset CHASE-DB1 Dataset EM Dataset	Semantic	✓	Kvasir-SEG Dataset: IoU: 0.815 Pre: 0.900 EM Dataset: IoU: 0.913 Pre: 0.952 2018 DSB: IoU: 0.856 Pre: 0.919 ISIC 2018: IoU: 0.802 Pre: 0.923
[47]	2018	UNet++	Cell (nuclei) segmentation	Microscopy images Colonoscopy videos Liver in CT scans Lung nodule	Semantic	✓	Cell nuclei: IoU: 0.907 Colon polyp: IoU: 0.300 Liver: IoU: 0.766 Lung nodule: IoU: 0.714
[48]	2024	SEAM-Unet++	Cell contour segmentation	FISH images (private data)	Semantic	⨯	IoU: 0.901
[49]	2024	YOLOv5 for detection and U-Net for segmentation	Cell detection and segmentation	Clinical Bacterial Dataset	Semantic	✓	MAP > 0.8
[50]	2024	csPWS-seg	Cell (nuclei) segmentation	csPWS microscopy images of HCT116 cells	Semantic	⨯	Median IoU: 0.780 DSC: 0.860
[51]	2023	GeneSegNet	Cell segmentation	Real dataset of human non-small-cell lung cancer (NSCLC) Real dataset of mouse hippocampal Area CA1 (hippocampus)	Instance	✓	IoU: gains of 5% to 15%
[55]	2024	Lightweight YOLOv4	Detecting malaria-infected red blood cells	MP-IDB, thin blood smear images obtained from MRC	Instance	⨯	ACC: 0.907
[56]	2024	CycleGAN model	Cell segmentation	DeepSea, LIVECell, Cell Tracking Challenge, Cellpose	Instance	✓	DSC: 0.853
[57]	2024	DaCSeg	Chromosome segmentation in metaphase cell images	985 Giemsa-stained microscopic metaphase images	Instance	✓	MAP: 0.841
[58]	2024	LKCell	Cell (nuclei) segmentation	PanNuke, MoNuSeg,	Instance	✓	PanNuke dataset: mPQ: 0.508 bPQ: 0.684 MoNuSeg: F1: 0.829 DSC: 0.839
[59]	2024	Self-supervised learning-enhanced Mask R-CNN	Segmentation and counting of MSCs	342 high-resolution bright-field microscopy images of mesenchymal stem cells (MSCs)	Instance	⨯	Pre: 0.884 Re: 0.867 DSC: 0.870
[60]	2024	CellViT	Detection and segmentation of cell nuclei	PanNuke, MoNuSeg, CoNSeP.	Instance	✓	PanNuke dataset: Pre: 0.850 Re: 0.830 F1: 0.840
[61]	2021	Mask R-CNN and Shape-Aware Loss	Cell segmentation	DIC-C2DH-HeLa dataset PhC-C2DH-U373 dataset	Instance	⨯	DICC2DH-HeLa IoU: 0.919 PhCC2DH-U373 IoU: 0.949
[62]	2019	C-LSTM with the U-Net	Cell Tracking	Fluo-N2DH-SIM DIC-C2DH-HeLa PhC-C2DH-U373x	Instance	✓	-
[63]	2019	Attentive neural cell instance segmentation method	Cell segmentation	644 neural cell images from a collection of timelapse microscopic videos of rat CNS stem cells	Instance	✓	IoU: 80.83
[70]	2023	DL based on cGANs	Cell segmentation	Salivary gland dataset Fallopian tube biopsy	Instance	⨯	Salivary gland dataset: fID: 46.61 training steps: 1800 steps Fallopian Tube Dataset fID: 56.45 training steps: 12,140
[71]	2018	SCWCSA	Cell segmentation	Dataset containing images of five cellular assays in 96-well microplates	Instance	⨯	ACC: 0.84
[72]	2019	Box-based method	Cell (nuclei) segmentation	neural cell dataset 2018 DSB	Instance	✓	Neural cell dataset: Pre: 0.880 IoU: 0.799 DSB: Pre: 0.718 IoU: 0.862
[73]	2023	Residual Attention U-Net	Cell and tissue segmentation	Bright-field transmitted light microscopy images	Both	⨯	MIoU: 0.953, MDSC: 0.975
[74]	2022	3DCellSeg pipeline	Cell segmentation	ATAS HMS LRP Ovules	Both	✓	ATAS: ACC: 0.956 HMS: ACC: 0.764 LRP: ACC: 0.747 Ovules: ACC: 0.822
[78]	2021	CS-Net	Cell segmentation	EPFL dataset Kasthuri++ dataset CPM-17	Both	✓	EPFL dataset: DSC: 0.994 JI: 0.990 Kasthuri++Dataset: DSC: 0.967 JI: 0.993 CPM-17: DSC: 0.883 JI: 0.711
[79]	2023	MSCA-UNet based on density regression	Cell counting	Synthetic bacteria (VGG) bone marrow (MBM) dataset human adipose tissue (ADI) dataset	-	⨯	Cell tracking: VGG MAE: 2.4 MBM: 8.0 ADI: 11.5 Cell detection VGG: Pre: 1.00 ± 0.00 Re: 0.917 ± 0.005 F1: 0.9538 ± 0.007 MBM: Pre: 0.901 ± 0.005 Re: 0.893 ± 0.004 F1: 0.894 ± 0.003 ADI: Pre: 0.981 ± 0.002 Re: 0.854 ± 0.0081 F1: 0.912 ± 0.006
[83]	2022	SAU-Net	Cell counting	VGG MBM ADI DCC MBC	-	✓	VGG: Pre: 0.999 MBM: Pre: 0.887 ADI: Pre: 0.885 DCC: Pre: 0.995 MBC: Pre: 0.925
[84]	2021	Concatenated fully convolutional regression network	Cell counting	Synthetic bacterial cells Bone marrow cells Colorectal cancer cells Human embryonic stem cells (hESCs)	-	⨯	Synthetic bacterial cells: MAE: 2.37 ± 2.27 Bone marrow cells MAE: 6.55 ± 5.26 Colorectal cancer cells: MAE: 29.34 ± 25.4 hESCs: MAE: 32.89 ± 26.35

On the other hand, in the context of instance cell segmentation, a Mask R-CNN-based framework proved robust, achieving high IoU values on diverse datasets. LSTM-UNet integrated convolutional LSTM with U-Net, achieving SOTA results on the Cell Tracking Challenge. GAN-based methods for instance cell segmentation utilized regular and conditional GANs, effectively simulating distribution, shape, and appearance of objects.

#### 4.1.2. Nucleus Segmentation

Table 3 offers a summary of the selected studies focused on nucleus segmentation, showcasing the range of methodologies and applications for this investigation.

In this section, we will investigate an examination of various studies, categorizing them based on their approaches to semantic and instance segmentation.

Starting with semantic segmentation, in [87], NucleiSegNet, a semantic architecture specifically designed for nucleus segmentation in H&E stained liver cancer histopathology images, was introduced. This DL architecture exhibited superior performance, as indicated by higher F1 and JI scores, in comparison to some recent SOTA models.

In [88], a recent network called SAC-Net was introduced for semantic nucleus segmentation on histopathology image datasets, utilizing point annotations. The network exhibited a highly competitive performance in cell nuclei segmentation across three public datasets.

In [89], the authors introduced GSN-HVNET, a model designed for semantic segmentation and classification. Experimental results showcased the superiority of the proposed model over other SOTA models such as Hover-Net [90], Micro-Net [91], DIST [92], and Mask R-CNN. GSN-HVNET demonstrated improvements in both segmentation and classification ACC while also maintaining high computational efficiency.

An effective method for semantic nucleus segmentation, FRE-Net, was introduced in [93]. The proposed approach demonstrated an outstanding performance across all four datasets, with DSC coefficients reaching 0.8563, 0.8183, 0.9222, and 0.9220 on the TNBC, MoNuSeg, KMC, and Glas datasets, respectively. Notably, the method exhibited superior boundary ACC and reduced instances of sticking compared to other end-to-end segmentation methods. These results underscore the capability of the FRE-Net method to outperform other SOTA segmentation methods.

In [94], the authors introduced Kidney-SegNet for semantic nucleus segmentation in histology images. The experiments showed that Kidney-SegNet exhibited very efficient computational complexity and memory requirements compared to existing SOTA DL methods.

In [95], the authors introduced AlexSegNet for instance nucleus segmentation. AlexSegNet is constructed upon the AlexNet model’s encoder–decoder framework. In the encoder section, it combines feature maps along the channel dimension to accomplish feature fusion. The decoder section employs a skip structure to integrate low- and high-level features, ensuring effective nucleus segmentation. The experimental findings demonstrated that AlexSegNet exhibited superior performance, particularly in terms of Re, Pre, and F1. For the 2018 DSB dataset, it achieved values of 0.931, 0.923, and 0.916, respectively. In the case of the TNBC dataset, it attained values of 0.542, 0.886, and 0.6688, respectively.

CNAC-Seg, proposed in [96], introduces a segmentation framework specifically designed for cervical nuclei in adherent cells, effectively addressing issues like overlapping nuclei and varying cell sizes. The CNAC-Seg model achieved a DSC of 0.886, an IoU of 0.815, and an MAE of 12.3, showcasing high ACC in densely populated scenarios. It excelled in delineating overlapping nuclei and improved boundary clarity compared to existing methods. Key components, especially the receptive field optimization module, enhanced segmentation accuracy. Overall, CNAC-Seg shows great promise for advancing cervical cell analysis in clinical diagnostics and research.

The proposed method in [97], FAFS-UNet, enhances the traditional UNet architecture by introducing two key modules to improve its skip connections: the Feature Aggregation (FA) Module and the Feature Selection (FS) Module. The FA Module aggregates features from the encoder and extracts local multi-scale information to provide comprehensive features for the decoder. Meanwhile, the FS Module selectively guides the decoder’s semantic recovery by focusing on the most relevant features. Experiments validated FAFS-UNet’s effectiveness, with improvements in segmentation performance, including a 2.10% IoU increase on the MoNuSeg dataset and a 1.83% IoU improvement on the CVC-ClinicDB dataset. The method also excelled in multi-organ segmentation tasks, particularly in preserving boundary information, surpassing other state-of-the-art algorithms.

The EMTL-Net model, introduced in [98], improves gland and nuclei segmentation in histopathology images by addressing common DL limitations. It features the Tweaked Residual Module (TRM), which enhances feature aggregation and reduces computational load by replacing the standard U-Net encoder with modified residual blocks. The Contextual Feature Decoding Module (CFDM) captures regional context, while the Contextual Feature Module (CFM) processes multi-scale objects for accurate segmentation of complex structures. The decoding path also incorporates long-range dependencies for precise boundary delineation. Evaluated through five-fold cross-validation on datasets such as Glas, TNBC, and MoNuSeg, EMTL-Net achieves a 4% increase in MIoU and a 2.6% improvement in MDSC compared to the LViT model, with gains of 5.3% and 3.8% over ConvUNeXt. Visual analyses on challenging MoNuSeg samples demonstrate EMTL-Net’s superior ability to capture intricate details and maintain boundary accuracy, signifying a notable advancement in segmentation performance and reliability for automated diagnosis in histopathology.

Additionally, various methodologies have been suggested in the context of instance segmentation.

In [99], authors introduce a DL model for real-time nuclei instance segmentation in histological images, combining U-Net and residual networks to improve accuracy and efficiency. By using a modified 2D convolutional layer, the model reduces computational complexity while preserving essential spatial features. Trained on the MoNuSeg-2018 dataset, it achieved an AJI of 0.7113 and an F1 of 0.9312, outperforming state-of-the-art models. With a processing speed of 41.667 frames per second, the model can segment up to 1000 whole slide images (WSIs) daily, making it highly suitable for real-time clinical applications.

In [100], the authors introduced ASW-Net for nucleus segmentation. The experiments were conducted using a Benchmark dataset, specifically the BBBC039 dataset [101], along with a ganglioneuroblastoma image set [102]. To assess the prediction performance of this method, ASW-Net, comparisons were made against CellProfiler [103], U-Net, and SW-Net (ASW-Net without attention gates), using ground truth as a baseline. The experimental results indicated that ASW-Net achieved satisfactory ACC in classification, even when confronted with an insufficient number of labeled training samples.

In [104], a pipeline called FPN with U-Net was introduced. It underwent evaluation and demonstrated superior performance compared to SOTA methods on two datasets, namely 2018 DSB and MoNuSeg.

In [105], a benchmark for instance nucleus segmentation was introduced. The authors conducted a comparative analysis of the segmentation effectiveness of five DL architectures and two conventional algorithms for segmenting nuclear images of immunofluorescence-stained samples. The DL architectures were categorized into two groups: U-Net architectures (U-Net, U-Net with a ResNet34 backbone (U-Net ResNet), U-Net based on transformed image representation (Cellpose)), and instance-aware segmentation architectures (Mask R-CNN, KG instance segmentation).

In [106], the authors introduced VRegNet, a Fully Convolutional Regression Network designed for nucleus detection in a cardiac embryonic dataset. This approach presented a combination of nuclei segmentation and centroid-regression networks, aiming to enhance the detection of nuclei in large 3D fluorescence datasets. It demonstrated high ACC in detecting centroids in both intact quail embryonic hearts and the mouse brain stem. Notably, this success was achieved even in tissues with clustered nuclei of diverse shapes, sizes, and fluorescent intensity. The performance of VRegNet was compared with different methods. The architecture demonstrated a good performance of 0.950, 0.935, and 0.942 in terms of Pre, Re, and F1.

In [107], the authors introduced RIC-Unet, a network designed for instance nucleus segmentation. RIC-Unet was compared with two traditional segmentation methods, CP and Fiji, as well as two original CNN methods, CNN2 and CNN3. Additionally, a comparison with the original U-Net was conducted using The TCGA dataset.

In [108], the authors introduced TSFD, a network designed for instance nucleus segmentation. This proposed network demonstrated superior performance compared to SOTA networks, including StarDist, Micro-Net, Mask R-CNN, Hover-Net, and CPP-Net, on the PanNuke dataset. The PanNuke dataset comprises 19 different tissue types and five clinically important tumor classes. TSFD achieved good mean and binary panoptic quality scores of 50.4% and 63.77%, respectively.

In [109], the authors introduced the NuClick network designed for interactive segmentation of objects in histology images. The applicability of NuClick was demonstrated across various datasets, including the gland dataset, nuclei dataset, and cell dataset.

Additionally, in [110], BAWGNet was introduced for instance nucleus segmentation. Comprehensive experiments conducted on three benchmark histopathology datasets (2018 DSB, MoNuSeg, and TNBC) showcased the exceptional segmentation performance of the proposed method, achieving a DSC of 0.908, 0.857, and 0.785, respectively.

Additionally, ref. [111] introduced ASPPU-Net, a model designed for segmenting instance nuclei. This architecture employs a modified U-Net with atrous spatial pyramid pooling. Experimental results demonstrated that incorporating the ASPPU-Net model with a concave point detection approach resulted in an improved ACC for delineating both individual and interconnected nuclei in histopathological images.

In [112], a region-based convolutional network was introduced to address nucleus detection and segmentation challenges. The proposed approach incorporates a GA-RPN module that integrates guided anchoring (GA) into the region proposal network (RPN) to generate candidate proposals optimized for nuclei detection. Additionally, a new branch is introduced to regress the IoU between the detection boxes and their corresponding ground truth, facilitating precise bounding box localization. To address challenges related to undetected adhered and clustered nuclei, a fusioned box score (FBS) is introduced and passed into soft non-maximum suppression (SoftNMS) to retain true positive candidate boxes.

The proposed method [113] introduces a hybrid attention-aware network for enhanced nuclei segmentation, combining spatial and channel attention mechanisms to improve focus on relevant spatial features and inter-channel relationships. The architecture includes an upsampling process for higher feature resolution, followed by a feature merging module to integrate local and global patterns. Morphological operations, watershed segmentation, and adversarial learning refine nuclei boundary delineation, while stain normalization ensures robust performance across varying staining conditions. The deep network utilizes dense layers and shortcuts to mitigate gradient vanishing and strengthen feature extraction. Evaluation results show significant accuracy improvements with scores of 0.960 for ACC, 0.874 for Re, 0.936 for Pre, 0.844 for IoU, and 0.865 for DSC, outperforming methods like CellSeg and Hover-Net. This framework not only advances segmentation precision but also supports pathologists with quantitative insights, marking a major step forward in digital pathology.

The DSCA-Net model proposed in [114] provides a rapid and efficient solution for automated cell segmentation in histopathological images. It combines a hierarchical feature extraction module with a streamlined external decoder network, effectively leveraging the unique morphological characteristics of cell nuclei. Tested on the PanNuke dataset, DSCA-Net surpassed advanced models like U-Net across various tissue types, achieving a PQ of 0.86 and an IoU of 0.80 for epithelial tissue, a PQ of 0.85 and an IoU of 0.78 for neoplastic tissue, and a PQ of 0.83 and an IoU of 0.76 for stroma tissue. These outcomes underscore DSCA-Net’s high segmentation accuracy across diverse tissue types, establishing it as a leading choice in tissue segmentation tasks.

The proposed Test Time Stain Normalization [115] (TTSN) method markedly improved histological image segmentation, raising the MDSC from 0.70 to 0.83 and the MIoU from 0.63 to 0.75 compared to the baseline. Additionally, TTSN achieved an MPre of 0.80 and an MRe of 0.85. On specific datasets, TTSN achieved a DSC of 0.76 for TNBC and 0.83 for NuInsSeg. By mitigating variability from staining differences, TTSN significantly enhanced segmentation accuracy across all metrics, with statistically significant improvements (*p* < 0.01). These results suggest that TTSN provides notable accuracy and robustness gains in histological image segmentation, advancing diagnostic efficiency in pathology workflows.

The study [116] presents a DL toolkit designed to improve 3D nuclei segmentation and quantitative analysis, featuring an iterative training strategy that refines segmentation accuracy. Initially, the model StarDistResNet_3Dnuc_initial was employed to segment weakly stained nuclei. These segmentations were then human-proofed to create a ‘gold ground truth’, which was used to train six sets of refined 3D ‘gold models’. Implemented in PlantSeg, Cellpose, and StarDist, the gold models demonstrated superior segmentation precision, with PlantSeg and StarDist-ResNet achieving the highest fivefold average Pre scores, while Cellpose models showed comparatively lower accuracy. This toolkit significantly enhances 3D nuclei segmentation, establishing a reliable foundation for future cellular and tissue analysis advancements.

The RANCA, proposed in [117], is a sophisticated method designed for accurate nuclear segmentation in tissue-cleared microscopy images, employing a regionally adaptive active learning strategy and high-level contextual heuristics to create a non-linear mapping from local image appearances to segmentation labels. It addresses the challenges of densely packed nuclei through an adaptive fine-tuning strategy, enhancing segmentation precision in complex scenarios. In comparative evaluations against established techniques like ilastik, U-Net, Attention U-Net, Mask R-CNN, and Cellpose, RANCA achieved the highest accuracy with a DSC of 0.9216, a low false positive rate of 9.47%, and an even lower false negative rate of 6.21%. In contrast, Cellpose, the best competitor, attained a DSC of 0.9069 but with a higher false negative rate of 16.14%. RANCA also recorded a JI of 0.8546, underscoring its robust capabilities, especially in low-contrast images and overlapping nuclei, thereby marking a significant advancement in cellular analysis for genetically manipulated animal models.

The experiments were conducted on two challenging public datasets designed to evaluate an algorithm’s generalization across different varieties. The empirical results showcase that the proposed method exhibits superior detection and segmentation capabilities compared to existing SOTA methods.

Alternative DL strategies, such as cGAN [118], have been put forward for instance nucleus segmentation. Experimental findings indicate that employing a cGAN trained with a combination of synthetic and real data can substantially enhance the ACC of nuclei segmentation in histopathology images.

Furthermore, a comprehensive evaluation framework was introduced in [119], aiming to measure ACC, identify types of errors, and assess computational efficiency. This framework was employed to compare DL strategies for nucleus segmentation in Fluorescence images with classical approaches.

In the referenced work [120], the authors introduced the CIA-Net, a deep neural network designed for nuclei instance segmentation. The paper introduces an Information Aggregation Module (IAM) that facilitates collaborative refinement of nuclei and contour details by leveraging spatial and texture dependencies through bi-directional feature aggregation. Additionally, a novel smooth truncated loss function is proposed to modulate the perturbation of outliers in loss calculation, enhancing the network’s focus on learning informative samples and improving generalization capability. Experimental validation on the 2018 MICCAI challenge of Multi-Organ-Nuclei-Segmentation demonstrates the effectiveness of CIA-Net, surpassing all other 35 competitive teams by a significant margin. The CIA-Net achieves a noteworthy F1 of 0.8485, outperforming other architectures proposed in the literature, CNN3, and PA-Net.

In [121], an effective approach is presented, introducing a bending loss regularized network tailored for nuclei segmentation in histopathology images. The bending loss is a key component, imposing penalties based on the curvature of contour points. Notably, it assigns higher penalties to points with large curvatures and smaller penalties to points with small curvature, mitigating the generation of contours that span multiple nuclei. The proposed method is rigorously validated on the MoNuSeg and showcases superior performance when compared to six SOTA approaches. The comparison involves six recently published approaches, namely FCN8, U-Net, SegNet, DCAN, DIST, and Hover-Net, using metrics such as AJI, Dice, RQ, SQ, and PQ scores. The proposed approach attains the highest overall performance when benchmarked against these methods on a public dataset. The efficacy of the proposed bending loss regularized network is evident in its accurate segmentation and localization of overlapped or touching nuclei, as validated on the MoNuSeg dataset.

In the referenced work [122], the authors introduced a novel instance-aware self-supervised learning framework for nuclei segmentation, aiming to eliminate the need for manual annotations in DCNNs. To assess the effectiveness of the proposed proxy task, the authors conducted experiments using the publicly available MoNuSeg dataset. The experimental outcomes highlight the substantial improvement achieved by the self-supervised learning approach in enhancing the ACC of nuclei instance segmentation. Notably, the self-supervised ResUNet-101 achieved a new SOTA average Aggravated JI of 0.706, showcasing the efficacy of the proposed method.

The paper cited as [123] introduces a Hematoxylin-aware CNN model designed for nuclei segmentation, eliminating the need for color normalization. Structured as a Triple U-Net, the model comprises an RGB branch, a Hematoxylin branch, and a segmentation branch. The proposed method is assessed on three nuclei segmentation datasets—MoNuSeg, CoNSeP, and CPM-17 dataset. Ablation studies are carried out to assess the efficacy of the Hematoxylin-aware model and to understand the impact of various loss configurations.

In the provided reference [124], the paper introduces the CPN framework designed for object instance segmentation using fixed-size representations based on Fourier Descriptors. CPN is flexible, incorporating various backbone networks and is trainable end-to-end. The CPN architecture comprises five fundamental building blocks, involving the generation of dense feature maps, object detection through a classifier head, and the creation of explicit contour representations via regression heads. In experimental evaluations on diverse datasets, the CPN framework demonstrates superior instance segmentation ACC compared to U-Net, Mask R-CNN, and StarDist. Particularly, CPN with local refinement achieves the highest scores across all datasets. The local refinement additionally enhances average F1, particularly for high thresholds, contributing to improved contour quality.

**Figure 9:**
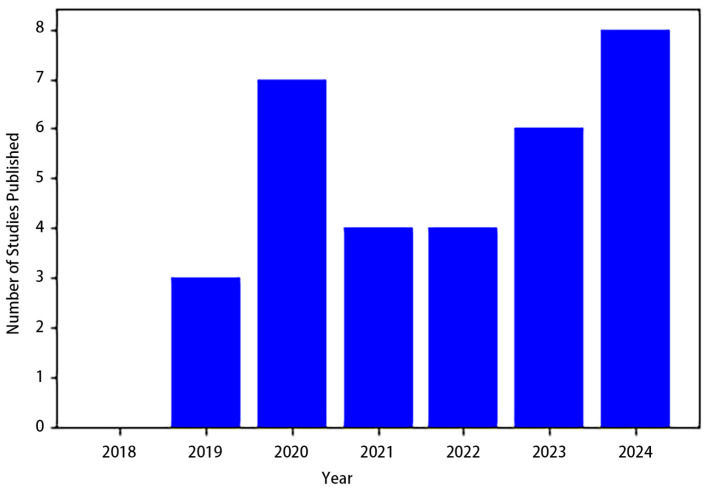
Number of studies published by year for nucleus segmentation.

The bar graph (see Figure 9) illustrates the annual study counts on nucleus segmentation from 2018 to 2024, revealing a minimal number of publications in the earlier years and a significant increase in 2023–2024. This trend reflects the growing interest and advancements in the field, driven by the exploration of diverse methodologies in nucleus segmentation. Notably, research in nucleus segmentation spans semantic and instance segmentation approaches. On the semantic segmentation front, innovative models like NucleiSegNet, tailored for liver cancer histopathology images, and DenseRes-Unet, effective on the MoNuSeg dataset, have achieved state-of-the-art results. Similarly, strategies like CIA-Net have excelled in nuclei instance segmentation, as demonstrated in the 2018 MICCAI challenge. Advances in instance segmentation include architectures such as FPN with U-Net, RIC-Unet, and Mask R-CNN, all of which outperform traditional methods and set benchmarks for efficiency and accuracy. Furthermore, techniques like TSFD have achieved notable results on the PanNuke dataset, highlighting the ongoing progress and innovation that likely contribute to the observed increase in publications. These advancements collectively push the boundaries of biomedical image analysis, reinforcing the relevance of the field.

**Table 3:** Summary of Nucleus Segmentation Studies in the Literature. ✓ indicates code availability, ⨯ indicates code not available.

Reference	Publication Year	Method	Task	Dataset	Instance/Semantic	Code Availability	Results
[87]	2021	NucleiSegNet	Nucleus segmentation	KMC liver Kumar dataset	Semantic	✓	KMC liver: F1: 0.835 JI: 0.720 Kumar dataset: F1: 0.813 JI: 0.688
[88]	2023	SAC-Net	Nucleus segmentation	MoNuSeg TNBC	Semantic	✓	MoNuSeg: IoU: 0.651 ± 0.04 DSC: 0.794 ± 0.04 TNBC: IoU: 0.636 ± 0.03 DSC: 0.776 ± 0.03
[89]	2023	GSN-HVNET	Nucleus segmentation and classification	CoNSeP Kumar CPM-17	Semantic	⨯	CoNSeP: DSC: 0.861 AJI: 0.602 PQ: 0.566 Kumar: DSC: 0.879 AJI: 0.635 PQ: 0.644 CPM-17: DSC: 0.899 AJI: 0.701 PQ: 0.683
[93]	2023	FRE-Net	Nucleus segmentation	TNBC MoNuSeg KMC Glas	Semantic	✓	TNBC: DSC: 0.856 MoNuSeg: DSC: 0.818 KMC: DSC: 0.922 Glas: 0.922
[94]	2021	Kidney-SegNet	Nucleus segmentation	Dataset of H&E images of kidney tissue TNBC Breast dataset	Semantic	✓	Kidney dataset: F1: 0.929 AJI: 0.868 TNBC breast dataset: F1: 0.824 AJI: 0.703
[95]	2023	AlexSegNet	Nucleus segmentation	2018 DSB TNBC dataset.	Semantic	⨯	2018 DSB: Re: 0.931 Pre: 0.923 F1: 0.916 TNBC dataset: Re: 0.542 Pre: 0.886 F1: 0.668
[96]	2023	CNAC-Seg	Cervical nuclei segmentation	ISBI Data, Herlev Data, private data	Semantic	⨯	ISBI Dataset: IoU: 0.858 ± 0.009 DSC: 0.923 ± 0.005 Herlev Dataset: IoU: 0.903 ± 0.008 DSC: 0.949 ± 0.012 Private data: IoU: 0.523 ± 0.017 DSC: 0.687 ± 0.015
[97]	2024	FAFS-UNet	Nucleus segmentation	MoNuSeg, CVC-ClinicDB	Semantic	✓	MoNuSeg dataset: IoU: 0.671 ± 0.017 DSC: 0.802 ± 0.012 ACC: 0.914 ± 0.005 Pre: 0.784 ± 0.012 Re: 82.47 ± 2.82 CVC-ClinicDB dataset: IoU: 0.876 ± 0.003 DSC: 0.933 ± 0.002 ACC: 0.987 ± 0.000 Pre: 0.934 ± 0.001 Re: 0.933 ± 0.003
[98]	2024	EMTL-Net	Nuclei segmentation	Glas, Triple Negative Breast Cancer (TNBC), MoNuSeg	Semantic	⨯	Glas: IoU: 0.869 DC: 0.923 Pre: 0.910 Re: 0.942 F1: 0.925 TNBC: IoU: 0.741 DC: 0.855 Pre: 0.849 Re: 0.875 F1: 0.863 MoNuSeg: IoU: 0.713 DC: 0.829 Pre: 0.795 Re: 0.869 F1: 0.837
[99]	2024	DL model for real-time segmentation	Nucleus segmentation	MoNuSeg 2018 challenge	Instance	⨯	AJI: 0.711 F1: 0.931
[100]	2022	ASW-Net	Nucleus segmentation	BBBC039 dataset Ganglioneuroblastoma image set	Instance	✓	DSC: 0.964 AJI: 0.902 DQ: 0.944 SQ: 0.916 PQ: 0.866
[104]	2020	FPN with a U-Net	Nucleus segmentation	2018 DSB MoNuSeg	Instance	✓	2018 DSB: AP: 0.608 IoU: 0.849 MoNuSeg: AP: 0.550 IoU: 0.808
[105]	2021	Benchmark of DL architectures	Nucleus segmentation	Annotated fluorescence image dataset	Instance	✓	U-Net ResNet: Re:0.829 Pre: 0.771 F1: 0.849, mDSC: 0.921 mJI: 0.859 AJI: 0.779
[106]	2021	VRegNet	Nucleus detection	Cardiac embryonic dataset	Instance	⨯	Pre: 0.950 Re: 0.935 F1: 0.942
[107]	2019	RIC-Unet	Nucleus segmentation	TCGA (The Cancer Genomic Atlas) dataset	Instance	⨯	AJI: 0.563 Average F1: 0.827
[108]	2022	TSFD-Net	Nucleus segmentation and classification	PanNuke dataset	Instance	✓	mPQ: 0.504 bPQ: 0.637
[109]	2020	NuClick	Nucleus and cell segmentation	Glas dataset MoNuSeg dataset Cell dataset	Instance	✓	MoNuSeg dataset: AJI: 0.912 DSC: 0.839 PQ: 0.838 Haus: 4.05 Cell dataset: AJI: 0.954 DSC: 0.983 PQ: 0.958 Haus: 7.45
[110]	2023	BAWGNet	Nucleus segmentation	2018 DSB MoNuSeg TNBC	Instance	✓	MoNuSeg: DSC: 0.857 IoU: 0.748 Pre: 0.799 Re: 0.924 Specificity: 0.663 ACC: 0.817 DSB: DSC: 0.854 IoU: 0.756 Pre: 0.874 Re: 0.865 Specificity: 0.971 ACC: 0.948 TNBC: DSC: 0.785 IoU: 0.619 Pre: 0.734 Re: 0.819 Specificity: 0.835 ACC: 0.829
[111]	2020	ASPPU-Net	Nucleus segmentation	TNBC; TCGA	Instance	⨯	TNBC dataset: DSC: 0.83 ± 0.02 ACC: 0.91 ± 0.04 IoU: 0.82 ± 0.04 TCGA: DSC:0.83 ± 0.05 ACC: 0.89 ± 0.02 IoU: 0.78 ± 0.04
[112]	2022	CNN	Nucleus detection and segmentation	2018 DSB MoNuSeg	Instance	✓	DSB: AP: 0.71 AR: 0.669 MoNuSeg: AP: 0.86.0 AR:0.55
[113]	2024	Hybrid attention-aware network	Nuclei segmentation	MoNuSeg, CoNSeP, PanNuke	Instance	⨯	MoNuSeg: ACC: 0.959 Re: 0.873 Pre: 0.935 MIoU: 0.843 DC: 0.864 PanNuke: ACC: 0.960 Re: 0.874 Pre: 0.936 MIoU: 0.844 DC: 0.865 CoNSeP: ACC: 0.939 Re: 0.853 Pre: 0.915 MIoU: 0.823 DC: 0.844
[114]	2024	DSCA-Net	Nuclei segmentation	PanNuke dataset, CoNSeP dataset	Instance	⨯	PanNuke: PQ: 0.357 MIoU: 0.524 CoNSeP: PQ: 0.581 MIoU: 0.425
[115]	2024	Generalized Stain Normalization Approach	Nuclei segmentation	MoNuSeg challenge, TNBC, CryoNuSeg, CPM-15, CPM-17, CoNSeP, NuInsSeg dataset	Instance	⨯	TNBC: DSC: 0.814 ± 0.002 AJI: 0.648 ± 0.002 PQ: 0.623 ± 0.004 CryoNuSeg: DSC: 0.790 ± 0.002 AJI: 0.520 ± 0.001 PQ: 0.492 ± 0.002 CPM-15 DSC: 0.757 ± 0.006 AJI: 0.546 ± 0.006 PQ: 0.508 ± 0.009 CPM-17: DSC: 0.823 ± 0.004 AJI: 0.643 ± 0.006 PQ: 0.643 ± 0.006 CoNSeP: DSC: 0.715 ± 0.001 AJI: 0.427 ± 0.3 PQ: 0.427 ± 0.003 NuInsSeg: DSC: 0.427 ± 0.003 AJI: 0.475 ± 0.004 PQ: 0.409 ± 0.006
[116]	2024	DL-based toolkit	3D nuclei segmentation and quantitative analysis	Fixed and live tissues, plant and animal Tissues, various nuclear stains	Instance	✓	Average Pre PlantSeg: 0.788 ± 0.019, StarDist: 0.7833 ± 0.017 Cellpose: 0.5196 ± 0.125
[117]	2024	RANCA	Nuclei segmentation in tissue-cleared microscopy images	Image set BBB038, available from the Broad Bioimage Benchmark Collection	Instance	⨯	DSC: 0.921 JI: 0.854
[118]	2020	cGAN	Nucleus segmentation	Annotations of 30 1000 × 1000 pathology images from seven different organs (bladder, colon, stomach, breast, kidney, liver, and prostate)	Instance	✓	AJI: 0.721 HD: 4.291 F1: 0.866
[119]	2019	DL Strategies	Nucleus segmentation	Fluorescence Images	Instance	✓	-
[120]	2019	CIA-Net	Nucleus	MoNuSeg dataset	Instance	⨯	AJI: 0.630 F1 0.845
[121]	2020	Bending loss regularized network	Nucleus	MoNuSeg	Instance	⨯	AJI: 0.641 DSC: 0.837 RQ: 0.760 SQ: 0.775 PQ: 0.592
[122]	2020	Instance-aware Self-supervised Learning for Nuclei Segmentation	Nucleus	MoNuSeg 2018 Dataset	Instance	⨯	AJI: 0.706
[123]	2020	Triple U-Net	Nucleus	MoNuSeg CoNSeP CPM-17	Instance	⨯	MoNuSeg: AJI: 0.584 ± 0.030 DSC: 0.817 ± 0.010 PQ: 0.544 ± 0.023 CoNSeP: AJI: 0.571 ± 0.016 DSC: 0.832 ± 0.012 PQ: 0.545 ± 0.017 CPM-17: AJI: 0.658 ± 0.017 DSC: 0.860 ± 0.011 PQ: 0.631 ± 0.013
[124]	2022	Contour Proposal Network	Cell detection Cell segmentation	NCB—Neuronal Cell Bodies BBBC039—Nuclei of U2OS cells BBBC041—P. vivax (malaria) SYNTH—Synthetic shapes.	Instance	✓	BBBC041 F1: 0.54 BBBC039 F1: 0.55

#### 4.1.3. Tissue Segmentation

Table 4 presents a summary of some studies about tissue segmentation identified in the literature. Our exploration begins with various approaches studied for semantic segmentation.

In [125], the authors introduced a novel network that integrates image processing techniques, including geometric augmentations and color augmentations, with a modified DL-based U-Net approach. The purpose of this combined approach is for semantic blood vessel segmentation.

The proposed model introduces in [126] a novel hierarchical hybrid architecture that integrates CNNs with the TransXNet block for glomeruli segmentation in renal histology imaging. Key features include a Token Mixing Module for blending global self-attention and local convolution, Input-Dependent Depthwise Convolution (IDConv) for adapting filters to input details, Overlapping Spatial Reduction Attention (OSRA) for better global context, and a Squeezed Token Enhancer (STE) for efficient cross-channel communication. The model’s downsampling and upsampling phases leverage TransXNet to extract features and restore spatial resolution. It outperforms existing models like U-Net and ResNet50, achieving 0.8513 MPA, 0.7680 MIoU, and 0.8280 MDSC. Ablation studies confirm optimal performance with minimal parameter increases.

The DRA-Net, proposed in [127], presents a novel approach to medical image segmentation, effectively addressing the limitations of conventional CNNs and Transformer-based models. It incorporates two innovative modules: the Dynamic Regional Attention Module, which adapts regions based on feature similarity for enhanced segmentation of targets with diverse shapes and sizes, and the Ordered Shift MLP Module, which fosters improved communication between feature groups to capture finer details. This architecture enables multi-range feature interactions and minimizes information loss during segmentation. DRA-Net showcased impressive performance, achieving an IoU of 0.8245 and a DSC of 0.902 on the Glas dataset, while also surpassing U-Net, UNet++, and MedT50 on the CoCaHis dataset.

The BGF-Net, proposed in [128], introduces an advanced method for medical image segmentation by integrating low-level and high-level feature guidance, focusing on boundary refinement through modules such as Contour Boundary Guidance (CBG), Spatial Boundary Guidance (SBG), and the Boundary Guided Filter (BGF). Evaluated across multiple datasets, BGF-Net consistently outperforms baseline models. On the ISIC 2016 dataset, it achieved a DSC of 0.9222, a JI of 0.863, and an ACC of 0.961. It also excelled in polyp segmentation on the CVC-EndoSceneStill dataset, with a DSC of 0.859 and an ACC of 97.0%. For gland segmentation on the Glas dataset, BGF-Net achieved a DSC of 0.860 and an MIoU of 0.754. These results highlight BGF-Net’s ability to enhance segmentation accuracy and handle complex lesion boundaries effectively.

The proposed CrossU-Net [129] method is designed to segment gastric precancerous lesions (GPL) by utilizing dual-modality images, combining RGB and hyperspectral data. The core of this method is a dual-stream U-Net architecture that extracts features from both image types, enhanced with a cross-attention mechanism to improve communication between spatial and spectral information. A Spectral-wise Siamese (SpeSiam) network is used for self-supervised pre-training on hyperspectral images, addressing the challenge of limited training data in medical imaging. Additionally, a distillation loss function aligns predictions from both RGB and hyperspectral branches, enhancing feature representation and model generalization. Experimentally, CrossU-Net achieved an ACC of 0.9653 and a DSC of 0.9162 for GPL segmentation, demonstrating its effectiveness in improving diagnostic accuracy for early gastric cancer detection.

The ResMTUnet [130] is a sophisticated model designed for precise segmentation of invasive carcinoma regions in breast tissue pathology images. By integrating vision transformers with convolutional neural networks (CNNs), ResMTUnet effectively captures both global and local features, while utilizing a multi-task learning strategy to boost segmentation and classification performance. A standout component is the MS-ResMTUnet, which employs conditional random fields to merge outputs from multiple scales, thereby enhancing segmentation accuracy in whole slide images (WSIs). When evaluated on the BRACS dataset, ResMTUnet achieved an impressive overall IoU of 0.8349, with a specific IoU of 0.762 for invasive carcinoma, alongside F1 scores of 0.892 and 0.905 for invasive carcinoma. These outcomes underscore ResMTUnet’s effectiveness in accurately identifying cancerous regions, highlighting its potential to improve automated diagnosis in digital pathology.

The paper [131] presents the Bi-BPT, an innovative medical image segmentation network that integrates transformers, CNNs, and an SAM encoder to effectively capture both long-range dependencies and local features. By utilizing an axial attention mechanism, Bi-BPT improves segmentation accuracy with limited training data while reducing computational demands. In evaluations on multiple datasets, it outperformed traditional CNN models like U-Net and Res-U-Net, achieving a PA of 0.928 and MIoU of 0.832 on the ISIC dataset. The model also excels in segmenting fine structures, surpassing competitors such as DAEFormer and MedT.

Additionally, the paper [132] introduces two models for lung cancer detection: TransSegNet, which utilizes transformer blocks for segmentation, achieving 0.996 accuracy, and MinClassNet, a seven-layer neural network for classification, reaching near-perfect accuracies of 1.000 and 0.9996. Overall, the proposed methods significantly enhance the accuracy and efficiency of lung cancer detection and segmentation.

HRD-Net [133] is an innovative high-resolution network designed for retinal vessel segmentation, effectively addressing challenges in feature map integrity through multi-scale representation. By employing high-resolution branches for detailed vessel capture and low-resolution branches for semantic feature recognition, HRD-Net reduces noise and enhances accuracy. The network features the Feature Extraction Convolution Module (FECM) with DCNv3 to capture complex vessel structures and the Adversarial Enhancement Module (AEM) to improve pixel-level accuracy. Evaluated against 14 state-of-the-art segmentation methods on the DRIVE, STARE, and CHASE_DB1 datasets, HRD-Net demonstrated outstanding performance, achieving high specificity scores (0.9833, 0.9862, 0.9874) and ranking third in sensitivity. It also recorded the highest overall ACC (0.9704, 0.9755, 0.9772) and F1 scores (0.8312, 0.8357, 0.8521), along with the top Area Under the Curve (AUC) scores (0.9885, 0.9914, 0.9928), highlighting its effectiveness in distinguishing blood vessels from background noise and confirming its superiority in detecting fine vessel details.

TSCA-Net [134] is a novel method that combines convolutional neural networks (CNNs) and transformer-based techniques to enhance gland and nucleus segmentation in medical imaging. Evaluated on five publicly available datasets, TSCA-Net outperformed models such as U-Net, ConvUNeXt, TransUNet, and Swin-Unet, particularly in gland segmentation with the Glas dataset, where it demonstrated clearer boundary delineation and superior alignment with ground truth annotations. In nucleus segmentation on the MoNuSeg dataset, characterized by small, low-contrast objects, TSCA-Net excelled at reducing false positives and accurately capturing shapes. It also achieved the highest DSC, IoU, and accuracy scores for both the Glas and MoNuSeg datasets. In breast lesion segmentation on the BUSI dataset, TSCA-Net achieved an F1 of 0.8846 and an IoU score of 0.808. Results from the Glas dataset showed good F1 and IoU scores of 0.931 and 0.8759, while in skin lesion segmentation on ISIC 2018, it attained scores of 0.9161 for F1 and 0.858 for IoU. Overall, TSCA-Net demonstrated remarkable versatility and robustness across various medical image segmentation tasks.

The Twin-SegNet [135] architecture enhances segmentation accuracy in complex medical images by integrating twin networks with advanced segmentation techniques. This model employs dual paths for feature extraction and a shared decoder, allowing it to learn from both original and augmented data, which improves robustness and generalization across different datasets. Evaluated across various medical imaging tasks, Twin-SegNet achieved impressive results: in breast lesion segmentation, it recorded an F1 score of 0.897 and an IoU score of 0.8254; for gland segmentation using the GLAS dataset, it attained an F1 score of 0.9422 and an IoU score of 0.8941; in skin lesion segmentation on ISIC 2018, it achieved F1 and IoU scores of 0.9245 and 0.8712; and in nuclei segmentation on MoNuSeg, it scored 0.8411 (F1) and 0.7234 (IoU). Additionally, for polyp segmentation, it recorded a low ASSD score of 0.30, demonstrating precision in capturing intricate polyp boundaries. Overall, these results underscore Twin-SegNet’s effectiveness in improving diagnostic accuracy across multiple medical imaging tasks.

The SAHIS-Net [136] is a novel method developed for segmenting cholangiocarcinoma (CCA) in microscopic hyperspectral images. It features a Spectral Attention (SA) module that adaptively weighs spectral information for improved feature extraction and an Attention-aware Feature Enhancement (AFE) mechanism to boost the model’s discriminative power. To combat the vanishing gradient problem, a multi-loss training strategy is implemented, facilitating efficient network training using Keras with a TensorFlow backend, the Adam optimizer, and binary cross-entropy loss. Evaluated with metrics like Mean Squared Error (MSE), Structural Similarity Index Measure (SSIM), and MIoU, SAHIS-Net demonstrated superior performance in CCA segmentation, achieving over 80% reduction in parameters and enhancements in training and inference times of 57.3% and 33.1%, respectively. Its efficiency and fast inference times position SAHIS-Net as a promising solution for practical applications in medical imaging.

A novel image segmentation technique, named RINGS [137], was introduced for the segmentation of prostate glands in histopathological images. Notably, the RINGS algorithm represents the first fully automated method capable of maintaining high sensitivity even in the presence of severe glandular degeneration. The proposed method aims to accurately detect prostate glands, providing valuable assistance to pathologists in making precise diagnoses and treatment decisions. The RINGS algorithm achieved a DSC of 0.9016.

In [138], the authors presented ER-Net, a method specifically designed for 3D vessel segmentation. Notably, the ER-Net incorporates a feature selection module that adaptively selects discriminative features from both an encoder and decoder simultaneously. This selective feature process aims to enhance the importance of edge voxels, leading to a significant improvement in segmentation performance. The effectiveness of the proposed method was thoroughly validated across four improvements in segmentation performance. The effectiveness of the proposed method was thoroughly validated across four publicly accessible datasets. The experimental results indicate that ER-Net generally outperforms other SOTA algorithms across various metrics.

The FineVess [139] framework is an advanced DL solution designed for the automatic extraction of glioma vasculature from confocal microscope images of cleared human tumor tissues. It employs a specialized 3D ResCBAM nnU-Net network to optimize segmentation in complex 3D images, integrating preprocessing and postprocessing techniques to enhance image quality and ensure vessel mask continuity. FineVess outperformed traditional vessel extraction methods and the baseline 3D nnU-Net, achieving a 2.27% improvement in the DSC, particularly excelling at identifying weak and discontinuous vessels. Its adaptability was demonstrated through fine-tuning on external datasets from various optical microscopy methods, leading to significant performance enhancements. Overall, FineVess serves as a powerful tool for accurately segmenting glioma vasculature, offering insights into tumor biology and treatment responses

In [140], the authors introduced MDC-Net, a technique designed for nucleus segmentation in digital pathology images. This method employs a deep fully convolutional neural network and integrates distance maps and contour information to effectively segment nuclei that may be touching. The results of the investigated experiments conducted on different datasets demonstrate the superiority of MDC-Net in terms of metrics such as AJI, F1, and HD.

In the context of instance segmentation, the work described in [90] introduces Hover-Net, a method designed for simultaneous nuclear segmentation and classification in histology images. Hover-Net capitalizes on the instance-rich details embedded in the vertical and horizontal distances from nuclear pixels to their centers of mass. This approach proves beneficial in distinguishing clustered nuclei and ensuring precise segmentation, particularly in regions with overlapping instances.

FastCellpose [141] is a highly optimized DL framework designed for the segmentation of glomeruli in whole mouse kidneys, significantly enhancing the original Cellpose model. It achieves a 7-fold increase in inference speed through a streamlined architecture that removes redundant features, and optimizes the conversion of vector field outputs to binary masks for a 16-fold speed boost. FastCellpose can segment an entire kidney dataset in approximately 3.5 h, achieving a DSC of 0.945 and surpassing other state-of-the-art methods with a DSC of 0.945 and an IoU of 0.894. It processes 2048 × 2048-pixel images in just 0.22 s for inference and 0.28 s for mask reconstruction, making it 12 times faster than previous techniques. Additionally, FastCellpose has proven effective in segmenting brain neuronal soma, demonstrating its versatility across different biological tissues.

**Figure 10:**
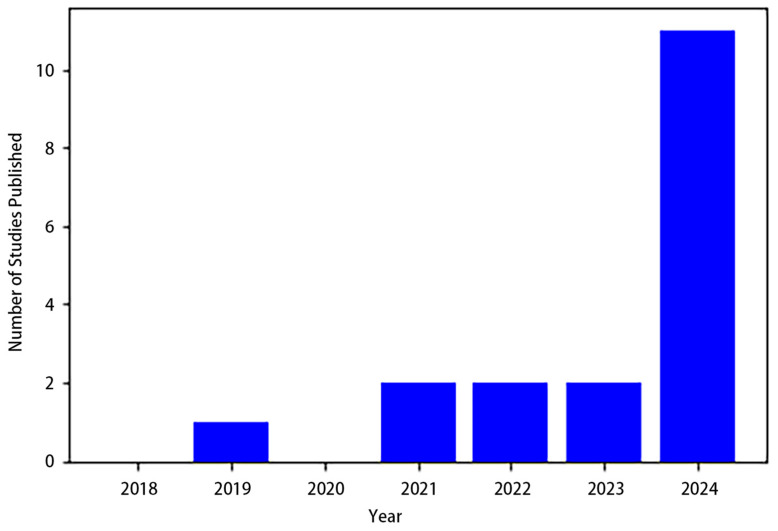
Number of studies published by year for tissue segmentation.

The bar graph (see Figure 10) highlights the annual number of studies published on tissue segmentation from 2018 to 2024. While early years saw minimal activity, there is a notable sharp increase in 2024, with 11 studies published. This trend reflects a growing research interest and advancements in tissue segmentation methodologies, driven by the demand for more accurate and efficient techniques. To sum up, the analyzed studies showcase significant progress in both semantic and instance segmentation. For semantic segmentation, methods like ER-Net stand out, specifically designed for 3D vessel segmentation. By incorporating adaptive feature selection, ER-Net enhances performance and precision. Similarly, Vessel U-Net integrates image processing techniques with a modified U-Net for semantic blood vessel segmentation, pushing the boundaries of semantic segmentation.

**Table 4:** Summary of tissue segmentation studies in the literature. ✓ indicates code availability, ⨯ indicates code not available.

Reference	Publication Year	Method	Task	Dataset	Instance/Semantic	Code Availability	Results
[125]	2022	Vessel U-Net model	Blood cell vessels	HAM10000 data set NIH studies R43 CA153927-01 CA101639-02A2	Semantic	⨯	ACC: 0.987 JI: 0.376 Pre: 0.574 Re: 0.521
[126]	2024	CNN-TransXNet	Segmentation of glomeruli in renal histology imaging	PAS-Stained Kidney Images	Semantic	⨯	PA: 0.851 IoU: 0.768 DSC: 0.828
[127]	2024	DRA-Net	Tissue segmentation in complex medical images	Glas, CoCaHis	Semantic	⨯	Glas: IoU: 0.824 DSC: 0.902 Re: 0.906 Specificity: 0.894 Pre: 0.901 CoCaHis: IoU: 0.7024 DSC: 0.824 Re: 86.95 Specificity: 0.889 Pre: 0.786
[128]	2024	BGF-Net	Tissue segmentations	ISIC 2016, CVC-EndoSceneStill, Glas	Semantic	⨯	ISIC 2016: DSC: 0.922 JI: 0.863 ACC: 0.961 SEN: 0.929 CVC-EndoSceneStill: DSC: 0.859 ACC: 0.970 SEN: 0.851 IoU: 0.753
[129]	2024	CrossU-Net	Gastric precancerous lesions segmentation	GPL dataset	Semantic	⨯	IoU 0.8463 DSC: 0.9162 PA: 0.9653
[130]	2024	MS-ResMTUnet	Invasive carcinoma regions in breast tissue segmentation and classification	BRACS dataset	Semantic	✓	Classification: ACC: 0.901 ± 0.009 F1: 0.892 ± 0.0087 Segmentation: IoU: 0.834 ± 0.012 DSC: 0.910 ± 0.009
[131]	2024	Bi-BPT	Tissue segmentation	MoNuSeg, MSD, COVID-19, Glas, ISIC	Semantic	⨯	COVID-19: PA: 0.958 IoU: 0.912 GLAS PA: 0.921 IoU: 0.853 MSD: PA: 0.915 IoU: 0.657 ISIC: PA: 0.928 IoU: 0.832 MoNuSeg: PA: 0.852 IoU: 0.682
[132]	2024	TransSegNet	Segmentation and classification of lung cancer	TSR-CRC	Semantic	⨯	ACC: 0.996 MIoU: 0.498
[133]	2024	HRD-Net	Retinal vessel segmentation	DRIVE dataset, The CHASE_DB1 dataset, The STARE dataset	Semantic	✓	STARE: ACC: 0.975 SE: 0.845 SP: 0.986 AUC: 0.991 F1: 0.835 DRIVE: ACC:0.970 SE: 0.837 SP: 0.983 AUC: 0.988 F1: 0.831 CHASE_DB1: ACC: 0.977 SE: 0.854 SP: 0.987 AUC: 0.992 F1: 0.852
[134]	2024	TSCA-Net	Tissue segmentation	MoNuSeg, Glas, TNBC, CHAOS-CT, CHAOS-MRI	Semantic	⨯	Glas: DSC: 0.886 ± 0.005 IoU: 0.807 ± 0.007 Pre 0.878 ± 0.009 Re: 0.909 ± 0.013 ACC: 0.890 ± 0.005 MoNuSeg: DSC: 0.802 ± 0.006 IoU: 0.671 ± 0.009 Pre 0.750 ± 0.022 Re: 0.869 ± 0.015 ACC: 0.906 ± 0.005 TNBC: DSC: 0.819 ± 0.010 IoU: 0.6975 ± 0.0123 Pre: 0.793 ± 0.016 Re: 0.860 ± 0.015 ACC: 0.949 ± 0.008
[135]	2023	Twin-SegNet	Tissue segmentation	Breast ultrasound lesion segmentation dataset, Glas dataset, ISIC 2018 dataset for skin lesion segmentation, MoNuSeg dataset for nuclei segmentation, CVC-ClininDB dataset for polyp segmentation	Semantic	⨯	BUSI: F1: 0.884 IoU: 0.808 Pre: 0.894 Rec: 0.902 ASSD: 0.010 Glas dataset: F1: 0.931 IoU: 0.875 Pre: 0.919 Rec: 0.948 ASSD: 0.013 ISIC 2018 dataset: F1: 0.916 IoU: 0.858 Pre: 0.927 Rec: 0.928 ASSD: 0.006 MoNuSeg dataset: F1: 0.817 IoU: 0.692 Pre: 0.786 Rec: 0.855 ASSD: 0.62
[136]	2024	SAHIS-Net	Segmentation of CCA in HSI	880 scenes of multidimensional images captured from choledochal tissues stained with hematoxylin and eosin.	Semantic	✓	MIoU: 0.616 ± 0.023 DSC: 0.616 ± 0.023 ASSD: 0.762 ± 0.017 AUC: 0.806± 0.013 ACC: 0.936 ± 0.014 Pre: 0.913 ± 0.004 Re: 0.785 ± 0.029
[139]	2024	FineVess	Segmentation of glioma blood vessels	3D glioma vessel data obtained by optical clearing, immunolabeling, and confocal microscope imaging, cerebrovascular volumetric data from two-photon fluorescence microscopy, 11 annotated vascular volumetric data imaged with light-sheet microscopy for analyzing cerebrovasculature of whole mouse brains	Semantic	✓	DSC: 0.761 Re: 0.771 Pre: 0.7728 ASD: 6.5661
[137]	2021	RINGS	Tissue (prostate) segmentation	Dataset of 1500 H&E-stained images of prostate tissue	Semantic	⨯	BalACC: 0.932 ± 0.068 Pre: 0.889 ± 0.135 Re: 0.935 ± 0.096 DSC: 0.901 ± 0.108
[138]	2022	ER-Net	3D vessel segmentation	Cerebrovascular datasets Nerve datasets: MIDAS-I, MIDAS-II	Semantic	✓	MIDAS-I: Sen: 0.921 Spe: 0.998 DSC: 0.861 AHD: 0.436 MIDAS-II: SEN: 0.713 SPE: 0.998 DSC: 0.687 AHD: 1.071
[140]	2021	MDC-Net	Tissue (nucleus) segmentation	DATA ORGANS DATA BREAST	Semantic	✓	DATA ORGANS: AJI: 0.595 HD: 6.774 F1: 0.823 DATA BREAST: AJI: 0.610 HD: 3.330 F1: 0.845
[90]	2019	Hover-Net	Tissue (nucleus) segmentation and classification	CPM-17 CoNSeP dataset Kumar	Instance	✓	Kumar: DSC: 0.826 AJI: 0.618 DQ: 0.770 SQ: 0.773 PQ: 0.597 CoNSeP: DSC: 0.853 AJI: 0.571 DQ: 0.702 SQ: 0.778 PQ 0.547 CPM-17: DSC:0.869 AJI:0.705 DQ: 0.854 SQ: 0.814 PQ: 0.697
[141]	2023	FastCellpose	Segmentation of glomeruli in whole mouse kidneys	Mouse Kidney Dataset	Both	⨯	DSC: 0.945 ± 0.016 IoU: 0.894 ± 0.028

In the realm of instance segmentation, Hover-Net and MDC-Net have been pivotal, addressing simultaneous segmentation and classification of nuclei in multi-tissue histology images. These contributions advance segmentation capabilities in complex tissue analysis. Further innovations include the RINGS algorithm, the first fully automated method for prostate gland segmentation in histopathological images, demonstrating exceptional sensitivity even in cases of significant glandular degeneration.

Collectively, these advancements underline the increasing impact of tissue segmentation research. The sharp rise in publications, particularly in recent years, mirrors the field’s evolution and growing significance in biomedical image analysis. This progress not only highlights the innovation in semantic and instance segmentation but also emphasizes the expanding scope of applications in tissue segmentation tasks.

#### 4.1.4. Common Benchmark Datasets

This subsection reviews well-known and widely benchmarked datasets commonly used in the field of MIS. As highlighted in previous subsections, various datasets have been employed across the literature to evaluate the performance of DL approaches. These datasets serve as critical benchmarks, enabling researchers to assess and compare the effectiveness of novel techniques in a standardized manner. Table 5 provides a comprehensive list of some of the most frequently used datasets for DL-based image segmentation.

### 4.2. RQ3

In this section, we will examine multiple papers that discuss various tools proposed in the literature for MIS and present a summarized overview of these studies in Table 6. The table includes details for each software/tool, such as the corresponding reference, microscopy image type, website, and associated task.

In [142], the paper introduces DeLTA 2.0, a Python-based workflow that employs DCNNs to analyze images of individual cells on two-dimensional surfaces, facilitating the quantification of gene expression and cell growth. Once trained, this workflow operates autonomously without requiring human input and demonstrates accurate processing of two-dimensional movies, effectively capturing spatial dynamics in a high-throughput manner. The algorithm leverages the U-Net neural network architecture for both segmentation and tracking models. The tracking model utilizes a sigmoid function as the final activation layer and employs a pixel-wise weighted binary cross-entropy loss function to generate a single grayscale output image, where 1s represent tracked cells, and 0s denote the background and cells that did not track to the input cell.

The DeepCell application [143] serves as a web-based tool and offers a scalable and cost-effective solution for conducting DL-powered cellular image analysis. This enables researchers to efficiently analyze extensive imaging datasets. Addressing the challenges posed by DL in biological image analysis, such as the requirement for extensive training data and substantial computational resources, the DeepCell Kiosk provides a solution. It enables the efficient allocation of resources and scalability according to the demand for data analysis, thereby diminishing analysis time and managing costs effectively.

Moving on, Cellpose [52] is another pipeline that facilitates nuclear and cytoplasmic segmentation, available as a web app or for local installation, complete with integrated annotation tools for training. Cellpose features a graphical user interface (GUI) with various preprocessing and postprocessing configuration options; however, command line usage is necessary for tasks like training or batch testing on user-specific data.

Another noteworthy tool is the DeepImageJ plug-in [144], providing a framework for testing models on researchers’ individual datasets. It offers an accessible solution designed for non-expert users to execute standard image processing tasks in life-science research. This is achieved by utilizing pre-trained DL models within the ImageJ platform like BioImage Model Zoo, enhancing user-friendly interactions. While it facilitates the user-friendly sharing of models, DeepImageJ currently only grants access to pre-trained models such as the BioImage Model Zoo, lacking a mechanism for users to train their models using their data. This limitation may pose challenges if existing pre-trained models prove insufficient.

**Table 6:** Tools for microscopy image segmentation.

Reference	Software/Tool	Microscopy Image Type	Website *	Tool Structure	Task
[142]	DeLTA 2.0	Time lapse microscopy data.	https://gitlab.com/dunloplab/delta https://delta.readthedocs.io/en/latest/	Web-based application	Cell segmentation and tracking
[143]	DeepCell	Fluorescence	https://deepcell.org/ https://github.com/vanvalenlab/kiosk-console	Web-based application Wrapper script Docker Container	Cell segmentation and tracking
[52]	Cellpose	Fluorescence Bright-field	https://www.cellpose.org/	Web-based application Jupyter notebook	Cell and nucleus
[144]	DeepImageJ	PCI	https://deepimagej.github.io/	ImageJ plug-in	Cell segmentation
[145]	CDeep3M	Light X-ray microCT electron microscopy	https://cdeep3m-viewer.crbs.ucsd.edu/cdeep3m_result/view/6447	Web-based application Google Colab Docker Container AWS cloud Singularity	Cell segmentation
[146]	DeepMIB	2D and 3D electron and multicolor light microscopy	http://mib.helsinki.fi https://github.com/Ajaxels/MIB2	MATLAB GUI	Cell
[147]	HistomicsML2	WSI	https://histomicsml2.readthedocs.io/en/latest/index.html https://github.com/CancerDataScience/HistomicsML2	Docker Container	Cell/nucleus/tissue
[148]	InstantDL	Bright-field CT scans	https://github.com/marrlab/InstantDL	Docker Container	Cell nucleus segmentation
[149]	NucleAlzer	Fluorescence Histology	www.nucleaizer.org	Web-based application	Nucleus segmentation
[150]	ZeroCostDL4Mic	Pseudo-fluorescence Bright-field	https://github.com/HenriquesLab/ZeroCostDL4Mic	Google Colab	Cell segmentation
[151]	Ilastik	Electron microscopy	https://github.com/ilastik	Python Script	Nucleus segmentation
[152]	Scellseg	Phase-contrast	https://github.com/cellimnet/scellseg-publish	GUI	Cell/tissue segmentation
[153]	DeepSea	Time-lapse	https://deepseas.org/software	MATLAB software tool	Cell segmentation
[154]	MIA	Phase-contrast Histology	https://doi.org/10.5281/zenodo.7970965	Python script	Image classification, object detection, semantic segmentation, and tracking
[155]	U-Net plugin	Fluorescence DIC Phase contrast Bright-field electron microscopy	https://lmb.informatik.uni-freiburg.de/resources/opensource/unet/	Caffe framework AWS cloud	Cell detection and segmentation
[156]	3DeeCellTraker	3D time lapse	https://github.com/WenChentao/3DeeCellTracker	Python script	Cell segmentation and tracking
[157]	StarDist	Bright-field Fluorescence	https://github.com/mpicbg-csbd/stardist	Docker Container	Cell/nucleus segmentation
[158]	SAM	Bright-field	https://github.com/computational-cell-analytics/micro-sam	Python script	Cell segmentation and tracking
[159]	BioImage Model Zoo	Microscopy images	https://bioimage.io/#/	Web-based application	LIVECell segmentation Cell segmentation Nucleus segmentation
[160]	NerveTracker	Serial block-face microscopy using 3D-MUSE	https://github.com/ckolluru/NerveTracker	Python-based software toolkit	Visualization of Nerve Fibers, Tracking Groups of Nerve Fibers, Microscopic Tractography, Interactive Editing
[161]	Biom3d	Confocal Microscopy Images, X-ray Microscopy Images, Electron Microscopy Images	https://github.com/GuillaumeMougeot/biom3d	Python Package Index	3D segmentation methods in various biological and medical imaging applications

*: accessed on 8 October 2024.

CDeep3M [145] stands out as a cloud-based tool specifically designed for image semantic segmentation tasks, offering pre-trained models tailored for electron micrographs. DeepMIB [146], on the other hand, is a deep learning-based image segmentation plug-in designed for both two- and three-dimensional datasets. It is integrated with the Microscopy Image Browser (MIB), an open-source MATLAB-based image analysis application for light microscopy and electron microscopy. DeepMIB allows users to load datasets, test pre-trained models, or even train a model using a graphical user interface (GUI).

HistomicsML2 [147] is an interactive segmentation tool designed specifically for Whole Slide Images (WSIs), tailored to enhance the ACC of semantic segmentation. This tool is dedicated to facilitating the segmentation process. HistomicsML2 is packaged as a Docker container, accessible through a web browser with a GUI. In the web-based GUI of HistomicsML2, biologists can annotate their data by dragging and dropping selected patches into corresponding classes. These annotations serve as the training data for a DL model employed in image segmentation. This process forms an active loop, wherein the DL model is trained using the initial annotations, applied to new data, and refined iteratively based on the ongoing annotations. Following each training step, regions of high uncertainty are displayed as a heatmap, enabling users to annotate these regions for further training to improve segmentation ACC. HistomicsML2 allows users to export results as HDF5 files, which can be further analyzed using other command line tools.

InstantDL [148] is a Python-based pipeline designed for segmentation and classification tasks. On the other hand, NucleAIzer [149] specializes in nuclear segmentation across various types and offers both web-based and local applications. Both tools utilize command line scripts for image processing, allowing users to configure parameters and execute tasks. While Cellpose [41] includes a GUI with preprocessing and postprocessing configuration options, command line usage is necessary for tasks like training or batch testing on data.

ZeroCostDL4Mic [150] is a compilation of readily available Google Colab notebooks designed for various image analysis tasks. This resource offers a range of Colab notebooks that facilitate the training of models across different tasks and image types.

Ilastik [151], an open-source toolkit for interactive ML, has introduced a beta version for image segmentation using pre-trained DL models. Although the installation process for utilizing neural networks with Ilastik is more intricate than typical usage, ongoing documentation efforts aim to simplify this procedure. Furthermore, the Ilastik team is actively working on enhancing capabilities for neural network training.

Scellseg [152] represents an adaptive pipeline tailored for cell segmentation algorithms. It features a style-aware cell segmentation architecture that leverages attention mechanisms and hierarchical information. This unique approach is crafted to optimize the extraction and utilization of style features. Scellseg has proven its state-of-the-art transferability, showcasing advancements over previous tools within the field.

DeepSea [153] stands out as annotation software, a MATLAB-based tool crafted for the cropping and labeling of cell and subcellular bodies in cell microscopy images. Specifically designed for segmentation and tracking tasks, DeepSea serves as an effective resource for annotating and processing microscopy data. Additionally, MIA [154] emerges as an open-source DL application tailored for microscopic image analysis. It encompasses three primary applications: segmentation, object detection, and classification.

The U-Net plugin [155] stands out as a DL software dedicated to cell counting, detection, and morphometry. Additionally, 3DeeCellTracker [156] introduces a DL-based pipeline designed for segmenting and tracking cells in 3D time-lapse images. Moreover, StarDist [157] demonstrates its efficacy in localizing cell nuclei using star-convex polygons, providing a superior shape representation compared to bounding boxes and eliminating the need for shape refinement.

The paper cited in reference [158] introduces SAM (Segment Anything for Microscopy), a tool derived from the vision foundation model known as Segment Anything for image segmentation. SAM enhances the model’s capabilities by developing dedicated models for microscopy data, thereby enhancing segmentation ACC. Additionally, it incorporates annotation tools for interactive segmentation and tracking, leading to accelerated data annotation compared to existing tools. The entire software is encapsulated within a unified Python library, encompassing both training and inference functionalities.

Lastly, The BioImage Model Zoo [159] is a centralized repository containing a diverse collection of pre-trained DL models specifically designed for bioimage analysis. This resource simplifies access to SOTA models across various applications, from image segmentation to object detection. Researchers benefit from the convenience of integrating these models into their projects, eliminating the necessity for extensive training efforts. This centralized hub fosters collaboration and accelerates progress in automated analysis techniques for intricate biological images.

NerveTracker [160] is a sophisticated Python-based software toolkit designed for visualizing and tracking nerve fibers in serial block-face microscopy images using ultraviolet surface excitation. It facilitates efficient analysis of nerve anatomy and responses to therapies through advanced image processing techniques, including modifications to algorithms like optic flow and structure tensor. With features such as interactive streamline visualization, manual editing, and optional DL segmentation for accurate fascicle identification, NerveTracker achieves a mean normalized Dice overlap metric exceeding 0.75, indicating high accuracy in nerve tract reconstruction. Its versatility allows application across various imaging methods, and the open-source code available on GitHub encourages further customization and development, marking a significant advancement in automated peripheral nerve analysis.

Biom3d [161] is a modular framework tailored for the development and hosting of 3D segmentation methods, specifically designed to enhance analysis in biological and medical imaging. Its architecture allows researchers to integrate and evaluate various segmentation algorithms on a wide range of datasets, including those from X-ray microscopy, electron microscopy, and confocal microscopy. This flexibility makes Biom3d a valuable tool for advancing research in computational biology, facilitating the comparison of different segmentation techniques, and providing robust solutions for complex imaging challenges. The framework is accessible as a public Python package, making it easy for the scientific community to implement and adapt it for specific research needs.

## 5. Discussion and Conclusions

The present SLR provides a comprehensive overview of methodologies and tools used in the field of Medical Image Segmentation (MIS). By synthesizing findings from 113 studies published between January 2018 and June 2024, this review highlights both the transformative potential of DL for MIS and the challenges that must be addressed to enhance its application in clinical and research settings. The findings cover a range of MIS levels—from cell and nucleus segmentation to tissue segmentation—each presenting unique challenges and requiring specialized techniques.

- Cell Segmentation

A majority of the reviewed studies focused on cell segmentation, reflecting the critical role of this task in various biological and medical applications. This choice is supported by their proven ability to deliver unparalleled ACC and operational efficiency in intricate image segmentation tasks. Remarkable strides are evidenced in specific methodologies such as the AS-UNet [40] algorithm, showcasing its exceptional performance on the BNS dataset. Equally notable is the SAU-Net [83], which extends the U-Net framework through the incorporation of self-attention modules, thereby elevating its capability to handle both 2D and 3D microscopy images. These advancements collectively contribute to the refinement of cell segmentation methodologies.

- Nucleus Segmentation

The literature revealed a growing emphasis on nucleus segmentation, considering its significance in pathological analysis and understanding cellular behavior. Several studies introduced novel architectures such as DOLG-NeXt [28], which outperformed SOTA U-Net and Transformer-based variants across multiple datasets. Equally important, the integration of attention networks, as exemplified in [100], showcased promising results in semantic nucleus segmentation, providing a foundation for further research in this direction.

- Tissue Segmentation

The review also addressed tissue segmentation, crucial for pathology and histology studies. ER-Net, proposed for 3D vessel segmentation, stood out for its adaptive feature selection module, significantly enhancing segmentation performance [138]. Furthermore, RINGS demonstrated a breakthrough in fully automated prostate gland segmentation [137].

- Integration of DL Tools

The integration of DL tools into existing platforms, as observed in DeepImageJ [144], offered researchers flexibility and accessibility. Nevertheless, limitations, such as the absence of a mechanism for user-specific model training, were identified. Additionally, tools like ZeroCostDL4Mic [150] provided readily available Google Colab notebooks for diverse image analysis tasks, democratizing access to DL capabilities. Moreover, a recent tool, SAM [158], showcases its efficacy in segmenting various microscopy data.

- Challenges and Future Directions

Even though microscopy image analysis has improved a lot, there are still challenges that show we need more focused research in the future. One big problem is not having enough labeled datasets, which slows down the progress of DL models in this area. In many cases, these datasets are also highly imbalanced, leading to biased models that may not generalize well to unseen data. Leveraging data augmentation techniques and synthetic data generation could help mitigating these issues. This emphasizes the importance of generating larger, more diverse, and well-explained datasets. Furthermore, the interpretability of increasingly complex models remains a crucial concern, demanding research into methodologies that enhance the transparency and comprehension of the decision-making processes within these models. Techniques such as explainable AI (XAI), which includes methods like saliency maps or class activation mapping (gradients, etc.), could be used to make these “black box” models more understandable, helping researchers to see which features influence decisions. It would also help in improving these models according to such study about features extracted throughout the neural networks.

Additionally, addressing the challenge of achieving generalizability across diverse microscopy images calls for the exploration of innovative techniques to adapt models to the inherent variations in imaging conditions. Transfer learning, where a model trained on one type of datum is fine-tuned for the use on another one, and domain adaptation methods may provide promising avenues for increasing the generalizability of DL models across various microscopy settings.

Looking ahead, a forward-thinking approach in future research should prioritize the establishment of standardized benchmarks, streamlining fair comparisons and systematic evaluations to ultimately drive progress in the resilience and applicability of microscopy image analysis techniques.

Our SLR has endeavored to provide a comprehensive synthesis of the available evidence on MIS. However, it is crucial to acknowledge the potential influence of publication bias on the observed results, as is inherent in the nature of SLRs. Specifically, studies with statistically significant results are often more likely to be published, while studies with negative or non-significant findings may be overlooked, which can skew the overall findings of this review. Addressing this bias in future research will require more transparency in the publication process and greater recognition of studies that report neutral or unexpected results. Similar to all SLRs, our findings could be susceptible to the preferential publication of studies exhibiting positive or statistically significant outcomes, potentially introducing a bias that may distort the overarching interpretation of the available evidence.

To sum up, this SLR provides a comprehensive analysis of the present status in the field of MIS, emphasizing the efficacy of DL methodologies in addressing intricate challenges. The integration of DL with classic image processing techniques could also offer hybrid approaches that combine the strengths of both methods. Noteworthy achievements underscore the increasing reliance on DL for precise and efficient segmentation tasks. Challenges identified underscore the need for ongoing research. As the field moves forward, further development of user-friendly tools and open-source software will be critical to democratizing access to advanced image analysis techniques for broader scientific and medical communities.

## Figures and Tables

**Table 1:** List of the abbreviations used in Section 4.

Abbreviation	Meaning	Abbreviation	Meaning
ACC	Accuracy	IoU	Intersection over union
AHD	Average Hausdorff distance	JI	Jaccard index
AJI	Aggregated Jaccard Index	KG	Keypoint graph
AS-UNet	U-Net with atrous depthwise separable convolution	LCM	Low-Cost Microscopes
ASSD	Average Symmetric Surface Distance	MAE	Mean Absolute Error
ASPPU-Net	Atrous spatial pyramid pooling U-Net	MAP	Mean Average Precision
ASW-Net	Attention-enhanced Simplified U-Net	McbUnet	Mixed convolution blocks
AWEL	Automatic weighted ensemble learning	MDC-Net	Multi-scale connected segmentation network with distance map and contour information
BalACC	Balanced accuracy	MDSC	Mean Dice Similarity Coefficient
BAWGNet	Boundary aware wavelet guided network	MIoU	Mean Intersection over union
BGF-Net	Boundary guided filter network	MoNuSeg	Multi-Organ Nuclei Segmentation
Bi-BPT	Bi-branch pyramid transformer	MPA	Mean Pixel Accuracy
CCA	Cholangiocarcinoma	MP-IDB	The Malaria Parasite Image Database
CIA-Net	Contour-aware Informative Aggregation Network	MPre	Mean Precision
cGAN	Conditional generative adversarial network	mPQ	Mean Precision Quality
C-LSTM	Convolutional Long Short-Term Memory	MRC	Malaria Research Centre
CPN	Contour Proposal Network	MRe	Mean Recall
csPWS-seg	Chromatin-sensitive Partial Wave Spectroscopic segmentation	MS-ResMTUnet	Multi-scale input model based on ResMTUnet
CS-Net	Cellular Segmentation Network	MSCs	Senescent mesenchymal stem cells
DSB	Data Science Bowl	PA	Pixel Accuracy
DCNN	Deep convolutional neural network	PCI	Phase contrast Image
DeLTA	Deep Learning for Time-lapse Analysis	PQ	Boundary Precision-Quality
DQ	Detection Quality	Pre	Precision
DSC	Dice Similarity Coefficient	RANCA	Regionally adaptive network via cascading architecture
DOLG	Deep orthogonal fusion of local and global	Re	Recall
DRA-Net	Dynamic regional attention network	Res-UNet-H	Residual U-Net for human sample
EMTL-Net	Explainable multitask learning network	Res-UNet-R	Residual U-Net for rat sample
ER-Net	Edge-reinforced neural network	ResMTUnet	Residual Mix Transformer U-Net
F1	F1-score	RIC-Unet	Residual Inception-Channel attention-U-Net
FARS	Fourier Adaptive Recognition System	RINGS	Rapid Identification of Glandural Structures
FCRN	Fully Convolutional Regression Network	SAU-Net	Self-Attention U-Net
fID	Frechet Inception Distance	SAM	Segment any model
FPN	Feature Pyramid Network	SBU-net	Saliency and Ballness driven U-shaped Network
FRE-Net	Full-region enhanced network	SCWCSA	Single-channel whole cell segmentation algorithm
GAN	Generative Adversarial Networks	SGAM-net	Saliency Guided Attention Module Network
Glas	Gland Segmentation in Colon Histology Images Challenge	SQ	Segmentation Quality
GPL	Gastric precancerous lesions	SSD	Single-shot detector
GRU	Gated Recurrent Unit	TCGA	Cancer genome atlas
H&E	Hematoxylin and Eosin	TSFD-Net	Tissue Specific Feature Distillation Network
HCM	High-Cost Microscopes	W-Net	Cascaded U-Net
HD	Hausdorff distance	WSI	Multiresolution whole slide images

**Table 5:** Overview of notable MIS datasets: This table presents information on different datasets, including the dataset type, the type of images or videos, and key characteristics such as the quantity of images and their dimensions.

Dataset	Image/Video Type	Description	Referenced By
ISBI 2012	Electron microscopy	Part of the ISBI 2012 challenge, this dataset focuses on segmenting neuronal structures in electron microscopy images. It includes 30 images (512 × 512) from the Drosophila ventral nerve cord, with annotations showing cells (white pixels) and membranes (black pixels). Images were resized to 128 × 128 for analysis.	[18,28,96]
2018 Data Science Bowl	Bright-field and fluorescence images	Comprises 670 images (256 × 256) of nuclei from bright-field and fluorescence microscopy.	[19,21,25,28,46,72,95,104,110,112]
Cell Tracking Challenge	2D and 3D time-lapse microscopy videos	Features 2D and 3D sequences of fluorescently stained nuclei/cells, bright-field, phase contrast, DIC microscopy videos, and synthetic videos with various shapes and motion.	[56,62]
MoNuSeg	2D microscopy images	The MoNuSeg challenge, part of MICCAI 2018, comprises a diverse set of 2D images from various biological samples, primarily focusing on the segmentation of nuclei in histological images. This dataset includes annotated images that serve as a valuable resource for training and testing segmentation models. By providing standardized images with detailed annotations, MoNuSeg supports the development and benchmarking of advanced algorithms in the field of medical image analysis, particularly for accurately identifying and segmenting cell nuclei in histopathological samples.	[21,57,59,87,92,96,97,98,103,108,109,111,112,114,119,120,121,122,130,133,134]
Colonoscopy (CVC-ClinicDB)	2D colonoscopy images	Provided by the 2015 MICCAI sub-challenge on automatic polyp detection, this dataset consists of 612 images (mostly sized 384 × 288) extracted from 25 different colonoscopy videos, showcasing various views of polyps. The ground truth consists of masks corresponding to the polyp regions in the images. Images were resized to 224 × 224 for processing.	[28,46,97]
ISIC challenge	2D Dermoscopic images	The ISIC Archive provides a large and diverse collection of skin lesion images, with over 25,000 annotated images from various skin conditions, including melanoma, benign nevi, and other types of skin cancer. The images come with associated metadata, including diagnosis and segmentation masks for training DL models.	[20,45,127,130,134]
PanNuke	2D histopathological images	The PanNuke dataset includes over 7000 annotated images from a variety of tissue types, specifically aimed at training models for the segmentation of nuclei in histopathological slides. It provides detailed annotations for various nuclei features and is used for benchmarking different segmentation algorithms.	[57,59,107,112,113]
CoNSeP	2D microscopy images	The CoNSeP dataset comprises 30 microscopy images with 10,000 annotated nuclei. It includes annotations made by multiple expert annotators to create a consensus for better accuracy. This dataset is particularly useful for training and benchmarking segmentation algorithms.	[59,88,89,112,113,114,122]
LIVECell	2D microscopy images	The LIVECell dataset includes 1000 images of live-cell imaging, primarily featuring different cell types under various conditions. Annotations are provided to aid in evaluating segmentation performance, focusing on challenging scenarios such as cell division and overlap.	[56,64]
TNBC	Histopathological images	The dataset comprises 50 images with 4022 annotated cells, including normal epithelial, myoepithelial, invasive carcinomatous cells, fibroblasts, endothelial cells, adipocytes, macrophages, and inflammatory cells. The number of cells per sample ranges from 5 to 293, with an average of 80 and a high standard deviation of 58. Annotations were conducted by three experts, including one pathologist and two trained research fellows, ensuring accuracy through a review process that involved consensus discussions for any disagreements.	[87,92,94,97,109,110,114,133]
Glas challenge	Histopathological images	The dataset for this challenge comprises 165 images obtained from 16 H&E-stained histological sections of stage T3 or T4 colorectal adenocarcinoma, with each section representing a different patient. Since these sections were processed in the laboratory at different times, the dataset exhibits significant inter-subject variability in both stain distribution and tissue architecture. The histological sections were digitized into whole-slide images (WSIs) using a Zeiss MIRAX MIDI Slide Scanner (Carl Zeiss AG, Oberkochen, Germany) at a pixel resolution of 0.465 µm.	[92,97,108,126,130,133,134]

## Data Availability

The systematic review and meta-analysis conducted in this study did not involve the generation or analysis of specific datasets. Instead, it utilized existing literature sources as the basis for analysis. All methodologies, search strategies, inclusion and exclusion criteria, as well as statistical analyses, are fully documented within the main body of this article.
